# Antigenicity in mice of a recombinant *Neisseria gonorrhoeae* MafA 2/3 protein

**DOI:** 10.1080/21505594.2025.2580086

**Published:** 2025-10-29

**Authors:** Michael M Girgis, Maria Victoria Humbert, Christopher J McCormick, Myron Christodoulides

**Affiliations:** a Neisseria Research Group, Molecular Microbiology, School of Clinical and Experimental Sciences, Faculty of Medicine, University of Southampton, Southampton, UK; b Department of Microbiology & Immunology, Faculty of Pharmacy, Manosura University, Manosura, Egypt

**Keywords:** *Neisseria gonorrhoeae*, MafA 2/3 protein, vaccine, bactericidal, murine, rabbit

## Abstract

There are no prophylactic vaccines for preventing the disease gonorrhea, caused by sexually transmitted infection with the Gram-negative pathogen *Neisseria gonorrhoeae*. In this study, we examined the antigenicity in mice of a recombinant rMafA 2/3 outer membrane protein (OMP). Mice were immunized with rMafA 2/3 with different adjuvants and delivery vehicles, which induced high levels of antibody that recognized the MafA 2/3 protein in i) antigen and OM-ELISA, ii) OM-western blots and iii) on whole bacteria examined with flow cytometry. Antisera to rMafA 2/3 in liposomes with monophosphoryl lipid A (MPLA) and with Zwittergent 3–14 ± MPLA, were bactericidal *in vitro* for homologous P9-17 (Allele 193) gonococcal strain (median 50% bactericidal titers of 256). Analysis of MafA 2/3 alleles among gonococcal isolates in the PubMLST database showed that ~50% and 26% of gonococci expressed Allele 90 and Allele 88-encoded protein respectively, and these proteins were identical bar one amino acid substitution. Murine antisera to Allele 193 rMafA 2/3 expressing strain P9-17 (~98% homology with Alleles 88/90 MafA 2/3 protein) showed bactericidal activity against heterologous strain FA1090 (Allele 88, 50% median titers from 4 to 64), but not to heterologous strain AR205 (Allele 90). By contrast, a rabbit anti-rMafA 2/3 serum was bactericidal for P9-17, FA1090, and AR205 (50% titers of 2048–4096), and inhibited significantly (*p* <0.05) the association of gonococci to human Chang conjunctival epithelial cells *in vitro*. These findings suggest that MafA 2/3 could be a promising OMP for further study as a component of future subunit gonococcal vaccines.

## Introduction

The Gram-negative bacterium *Neisseria gonorrhoeae* causes the sexually transmitted disease gonorrhea [1]. The organism is an obligate human pathogen that is transmitted primarily through sexual contact and targets the mucous membranes of the reproductive tract, causing urethritis and painful discharge in men and a mucopurulent cervicitis in women. However, in approximately 10–25% of untreated women, the bacteria can ascend into the upper reproductive tract causing pelvic inflammatory disease, which can leave patients with long-term and/or permanent chronic pelvic pain, fallopian tube damage, endometritis, ectopic pregnancy, and infertility [2]. Disseminated Gonococcal Infection (DGI) can result from infection of other mucosal and internal soft tissue body sites, including colonization of the ocular mucosa in neonates that is known as gonococcal *ophthalmia neonatorum*/conjunctivitis [3]. Co-infection with other sexually transmitted disease pathogens, e.g. HIV, *Treponema pallidum* (syphilis), *Trichomonas,* and *Chlamydia*, is common. Gonococci are also known to increase HIV transmission and infection [4]. There are an estimated 87 million new cases of gonorrhea reported annually [5], with the highest incidences in the Development Assistance Committee list of Official Development Assistance (ODA) least developed and low-to-middle-income countries. A significant number of infections are asymptomatic, and thus the true burden of infection is considerably higher.

Gonococci have rapidly developed resistance to every antibiotic class ever introduced since the 1930s (including sulfonamides, penicillins, tetracycline, and ciprofloxacin) with the World Health Organisation (WHO) classifying *N. gonorrhoeae* as a “High Priority” pathogen for the research and development of new antimicrobials and vaccines. Over the past decade, there have been increased reports of globally circulating multi-antibiotic resistant gonococci that are difficult to treat, with the first reports in 2016–2018 in the UK of failures to treat pharyngeal gonorrhea with ceftriaxone monotherapy and dual ceftriaxone-azithromycin therapy [6]. In recent years, only solithromycin, zoliflodacin, and gepotidacin have been developed as new antibiotics and evaluated clinically for treating uncomplicated gonorrhea. A recently concluded trial showed that solithromycin (1000 mg dose) was not a suitable alternative to ceftriaxone [7], further highlighting the limited treatment options underscoring the need to develop a prophylactic vaccine for long-term control. However, gonococcal vaccine development has been hampered by several challenges, including (i) the pathogen’s remarkable adaptability, which complicates the process of selecting suitable antigens, (ii) the absence of a natural and memory immune response after infection, resulting in a lack of known correlates of protection and (iii) the lack of a cost-effective animal model that truly mirrors all aspects of gonococcal transmission, infectivity, and disease development.

Gonococcal vaccine discovery has used traditional microbiological/proteomic/bio-informatic approaches to identify putative antigens that could be incorporated. Key vaccine strategies include outer membrane (OM) vesicles (OMVs), recombinant proteins, epitope-based peptides, and potentially mRNA and viral vector platforms [8–10]. In a recent immuno-proteomics study of sera collected from individuals with uncomplicated gonorrhea and reacted with a heterologous laboratory strain P9-17, we identified several proteins that showed increased reactivity with the sera and not with control sera from individuals with no history of gonorrhea [11]. One of these proteins was the putative Adhesin MafA 2/3, a SPII OM lipoprotein of 312 amino acids and molecular weight of ~33.8 kDa. The Maf proteins are a family of OM/secreted toxins in pathogenic *Neisseria* species [12] and recent studies showed that the *N. meningitidis* MafA protein formed oligomers in the OM. A protein of ~36kDa, which may be a MafA protein, was postulated to function as a glycolipid-binding adhesin, interacting with gangliotriosylceramide GgO3 and gangliotetraosylceramide GgO4 receptors expressed on human endocervical cells [13]. MafA 2/3 (NGO1393/1584) exhibits 97% identity to the product of the *mafA1* gene (AAF62309.1) of *N. meningitidis* MC58. Baarda et al [14] reported that *N. gonorrhoeae* strain FA1090 possessed four copies of MafA: two pairs of identical loci, designated MafA 1/4 (NGO1067/1972) and the MafA 2/3 (NGO1393/1584). Their data suggested that the two MafA subtypes were evolutionarily distinct, and the alleles associated with each subtype were closely related [14]. In strain P9-17, MafA 1/4 NGO1067 and NGO1972 are annotated as NEIS2083 in the PubMLST/*Neisseria* database and MafA 2/3 NGO1393 and NGO1584 as NEIS0596. In our immuno-proteomics study, patient sera recognized the MafA 2/3 NEIS0596 protein and NGO0225, the polymorphic toxin MafB class 2 protein. MafB is a toxin secreted by pathogenic *Neisseria* spp [12]. via the novel MafA protein secretion pathway [15]. In meningococci, MafB is a fratricidal toxin that can inhibit the growth of strains that do not produce corresponding immunity protein MafI [16]. In the current study, we focused on NEIS0596 and ignored NEIS2083, as the latter is a truncated form of the former.

A variety of different adjuvants and delivery vehicles are available for gonococcal vaccine antigens, including for rMafA 2/3, e.g. adsorption onto aluminum hydroxide (Alum) and incorporation into liposomes and Zwittergent micelles, the latter two with and without the addition of the Toll-*Like* Receptor 4 agonist monophosphoryl lipid A (MPLA). Alum has been the most widely used adjuvant with human vaccines for decades, and although the exact mechanism of action is not entirely clear, it favors the production of a Th2 response in mice characterized by increases in IgG1 antibody and decreases in IgG2a antibody [17]. Liposomes are increasingly used in vaccine formulations, e.g. AS01 (liposomal MPLA + QS-21) that is used in the Shingrix (herpes zoster vaccine), Mosquirix (RTS,S/AS01 for malaria) and Arexvy (RSV F-protein vaccine) vaccines [18]. They are generally safe and highly versatile and enable the incorporation of antigens into their membranes or encapsulation or surface linkage. Liposomes induce Th2-biased immune responses to antigens in mice, but varying liposomal size, composition, and the encapsulation of exogenous adjuvants can modulate the response. Zwittergent 3–14 is not an adjuvant in the classical sense, but detergent micelles have been used to solubilize *Neisseria* outer membrane proteins [19–22], and in some cases to generate immune responses with the incorporation of exogenous adjuvants [23–26]. Several studies with experimental bacterial and parasite vaccines have shown that MPLA is a potent inducer of Th1-type immune responses in mice, primarily though TLR4 activation [27]. Moreover, liposomes and detergent micelles that incorporate MPLA can skew the response toward Th1, with an increase in IgG2a antibody and a decrease in IgG1 antibody [28]. Thus, we tested the hypothesis that a recombinant (r)MafA 2/3 protein used with different adjuvant-delivery systems was antigenic in mice and could induce antibodies bactericidal for gonococci *in vitro*.

## Methods

### Bacterial strains, vectors, and growth conditions

*Neisseria gonorrhoeae* strain P9, a 1B-26 serovar isolate (ND: P1.18–10,43: F1-26: ST-1926), was originally isolated from a patient with gonococcal prostatitis [29]. The phenotype of derived P9-1 is Pil^−^Opa^−^ and P9-17 is Pil^+^Opa_b_^+^. Both have been used extensively in studies of gonococcal pathogenesis and for vaccine development. Strain FA1090 was obtained from the American Type Culture Collection (ATCC 700,825). The panel of 50 *N. gonorrhoeae* isolates assembled by the Centers for Disease Control and Prevention (CDCP) in collaboration with the Food and Drug Administration (FDA) was also obtained (Antibiotic/Antimicrobial Resistance Isolate Bank, https://www.cdc.gov/antimicrobial-resistance/php/public-health-strategy/index.html). Gonococci were grown on supplemented GC-agar plates [30] incubated at 37°C in an atmosphere containing 5% (v/v) CO_2_.

Gonococci were also grown in the presence of cytidine-5′-monophospho-N-acetylneuraminic acid (CMP-NANA), as described previously [26]. Briefly, 1 ml of a 1 mg/ml solution of CMP-NANA – sodium salt (Merck, UK) was sterilized by filtration, spread onto a surface of a 20 ml GC agar plate, and allowed to diffuse into the agar to give a final concentration of 50 µg/ml. Bacteria were grown as a lawn on the CMP-NANA plates for 16 h at 37°C in an atmosphere containing 5% (v/v) CO_2_, and then harvested and immediately used in serum bactericidal assays as described below.

The Geneart pMA-T plasmid (ThermoFisher Scientific, Paisley, United Kingdom) was used for cloning in *Escherichia coli* DH5α strain (Invitrogen, Monmouth, UK) and pET-22(+) vector and pHYRSF53-SUMO-tag vector [31] were used for recombinant protein expression in *E. coli* BL21(DE3)pLysS and *E.coli* BL21(DE3)/Rosetta strains. *E. coli* strains were grown at 37°C and 16°C on Luria Bertani (LB) agar and in LB broth for cloning and expression. When required, 100 μg/ml of ampicillin, 50 μg/ml of kanamycin, and 30 μg/ml of chloramphenicol were added for cloning and expression of recombinant protein.

### Preparation of gonococcal whole cell lysates and outer membranes (OM)

To prepare whole cell lysates, bacteria grown on GC agar plates were suspended in 500 µL of PBS and sonicated with a MSE Soniprep 150 (5 cycles of 20 seconds on and 20 seconds off). Bacteria were also grown in the presence of deferoxamine mesylate salt (Desferal, Merck, Gillingham, Dorset, UK) to deplete iron and promote the expression of proteins involved in iron uptake [11]. OM of strains P9-17, FA1090 and 12CFX_T_039 (AR205) were prepared by extraction in 0.2 M lithium acetate, pH 5.8 buffer with stirring with glass beads for mechanical disruption, as described previously [32,33]. The protein content of lysates and OM preparations was estimated with a Bicinchoninic Acid (BCA) assay (Pierce, ThermoFischer, UK), and all preparations were stored at −20 °C until required.

### In silico tools

*PubMLST*. Diversity and conservation of MafA 2/3 (NEIS0596, NGO1393/1584) was examined *in silico* among gonococcal isolates in the http://pubmlst/Neisseria.org database [34]. Protein amino acid sequences were aligned with Clustal Omega Multiple Sequence Alignment tool (Clustal Omega <EMBL-EBI). A putative structure of the MafA 2/3 and MafA 1/4 protein was generated with Swiss Model based on an AlphaFold DB model (UniProt A1KVQ6) [35].

### Cloning and expression of MafA 2/3 (NEIS0596) gene in E. coli

The mature *mafA 2/3* gene (NEIS0596) excluding the leader sequence was engineered *in silico* into a pMA-T plasmid (GeneArt, ThermoFischer, UK). The recombinant plasmid was transformed first into chemically competent *E. coli* DH5α for propagation, extracted from the bacterial cells and then digested using BamHI/HindIII restriction enzymes (Promega, Southampton, UK). For ligation of the insert gene with the expression vector (pET22(+) and pHYRSF53-SUMO), both DNAs were digested with the same restriction enzymes (BamHI/HindIII) and a molar ratio of insert/vector of 3:1 was used. The ligation reaction mixture consisted of 10 ng of vector, an appropriate amount of insert, 1 μl (3 U) of T4 DNA ligase (Promega, UK), and 5 μl of 2× rapid ligation buffer (Promega, UK). The mixture was incubated overnight at 4°C then transformed into competent *E. coli* strain DH5α (Invitrogen, UK) with selection on LB-ampicillin agar plates. Colonies were selected, plasmids purified and screened and then transformed into competent *E. coli* expression cells. Transformants were selected on LB agar plates containing ampicillin (50 μg/ml) or chloramphenicol (30 μg/ml). Pilot expression experiments were done to ensure the best expression conditions from both plasmids for the rMafA 2/3 protein by using two types of *E. coli* cells (*E. coli* BL21(DE3)/pLysS and *E. coli* BL21(DE3)/Rosetta). Protein induction was done by adding isopropyl-β-d-thiogalactopyranoside (IPTG) to a final concentration of 0.5 mM and 1 mM, and the culture was allowed to grow in a shaking incubator (Incu-Shake, SciQuip, Rotherham, UK) at different temperatures and times (i.e. 16°C or 18°C overnight, 25°C overnight and 37°C for 4 hours).

After pilot studies, a high yield of purified recombinant MafA 2/3 protein was generated as follows: *E. coli* BL21 (DE3) pLysS, carrying the recombinant MafA 2/3 plasmid pHYRSF53-SUMO was grown in 2 L of LB broth. Induction occurred at an optical density (OD) λ600 nm of 0.6–0.8 and the recombinant protein induced by adding IPTG to final concentration of 0.5 mM and allowed to grow overnight at 16°C. The bacterial cell pellet was harvested by centrifugation and suspended in lysis buffer (50 mM sodium phosphate monobasic monohydrate, 300 mM sodium chloride, 5 mM imidazole, pH8) (10 ml/g pellet). The bacterial suspension was then subjected to probe sonication (Soniprep 150; MSE, UK) on ice to release soluble rMafA 2/3 protein, using 20 seconds bursts, 5–10 times. The supernatant containing soluble protein was obtained after centrifugation and passed through a 0.45 μm filter. The lysate was then centrifuged at 10,000 × *g* for 30 minutes at 4°C.

### Purification of rMafA 2/3 protein

rMafA 2/3 protein was purified using Nickel-NitriloTriacetic Acid (Ni-NTA) metal-affinity chromatography (Cube Biotech, Monheim, Germany) under native conditions. Briefly, soluble protein was stored with nickel resin for 1 hour at 4°C and subsequently passed through a gravity flow column. Unbound proteins were washed using washing buffer (50 mM sodium phosphate monobasic monohydrate, 300 mm sodium chloride, 25 mm imidazole, pH8), and bound rMafA 2/3 protein was eluted using elution buffer (50 mM sodium phosphate monobasic monohydrate, 300 mm sodium chloride, 250 mm imidazole, pH8). The purified protein was assessed for purity and specificity by SDS-PAGE and western blot. The purified protein was then dialyzed into 10 mM HEPES buffer at pH 6.8. SUMO protease enzyme (also known as U1p) was added into the purified SUMO-fused protein with a ratio of 1:200 and incubated with the protein during the dialysis process to remove the SUMO tag from the recombinant fusion protein. The 6xHis-SUMO tag and SUMO protease enzyme were removed from the dialyzed protein solution by using 1 mL of 50% Ni-NTA PureCube His affinity agarose suspension (Cube Biotech). The purified protein without SUMO tag and SUMO protease enzyme were collected in the flow through. The protein was then concentrated using Amicon Ultra-15 centrifugal concentrator unit (Merck Millipore, Gillingham, Dorset, UK). The protein concentration was determined by BCA assay (Pierce, Thermo-Scientific, UK). A 6x HIS monoclonal epitope tag antibody (Invitrogen UK) was used in western blot at a 1/5000 dilution to identify expressed HIS-tagged recombinant protein prior to cleavage.

### Preparation of adjuvants and delivery vehicles for rMafA 2/3

#### Incorporation of rMafA 2/3 into liposomes

Liposomes were prepared using the dialysis-sonication method, as previously described [36]. Briefly, l-α-phosphatidylcholine and cholesterol (Merck) were combined in a molar ratio of 7:2 to a total amount of 20 mg and dissolved in 3 ml of chloroform within a nitric acid (70% v/v) washed glass round-bottom flask. The solvent was then removed under vacuum at 25°C with rotation (Rotavapor R110, Buchi, Newmarket, UK) to form a uniform lipid film. Simultaneously, a solution containing purified rMafA 2/3 (0.5 mg) and 100 mg of octyl β-d-glucopyranoside (Merck) were combined and dissolved in 2.5 ml of 10 mM HEPES (Merck) buffer, pH 7.2, and the solution was incubated at room temperature for 3 hours. The lipid film was dissolved in this protein solution and incubated at room temperature for 1 hour. The detergent-protein mixture was extensively dialyzed against 2 L of PBS containing thimerosal (1/10,000, w/v) for 72 hours at 4°C with twice daily changes of buffer and then subjected to sonication (MSE Soniprep 150 sonicator; 15 micron for 20 to 30 bursts of 60 seconds each) to induce vesicle formation. Any insoluble material was removed by centrifugation at 1,000 × g for 10 minutes. Liposomes were also prepared containing the adjuvant monophosphoryl lipid A (*S. enterica* serotype Minnesota MPLA, Merck) at an adjuvant/protein ratio of 1:1 (0.5 mg:0.5 mg). Control liposomes, both with and without protein and with or without MPLA, were also prepared. Aliquots of all liposome preparations were stored at −20°C until required.

#### Incorporation of rMafA 2/3 into Zwittergent micelles

Mixtures of rMafA 2/3 protein and Zwittergent were prepared in PBS, incorporating rMafA 2/3 (1.4 mg/mL stock) at a final concentration of 0.5 mg/ml protein and Zwittergent 3–14 (Calbiochem, Nottingham, UK) at 0.08% (w/v). These mixtures were prepared both with and without MPLA (0.5 mg/ml suspended in PBS) and incubated overnight at room temperature. For immunization, the solutions were further diluted with sterile saline (0.9% w/v NaCl in ultra-high-quality water) to achieve a final rMafA 2/3 concentration of 200 μg/ml. Control micelle preparations were also produced, excluding rMafA 2/3 and/or MPLA. Aliquots of all micelle preparations were stored at −20°C until needed.

#### Adsorption of rMafA 2/3 to Al(OH)_3_

A 2.0% suspension of aluminum hydroxide [Al(OH)_3_; alum gel adjuvant Alhydrogel, Invivogen, Toulouse, France) was used to adsorb rMafA 2/3 protein. A mixture was made to immunize 7 mice (excess) containing 140 μg of rMafA 2/3 (1.4 mg/mL stock) in a final volume of 350 μl and mixed 1:1 with 350 μl of alum. The protein was adsorbed by thorough mixing overnight at 4°C on an angled rotary mixer. The preparations were immediately used for immunization. Control alum was prepared without antigen.

#### rMafA 2/3 in saline

rMafA 2/3 (1.4 mg/mL stock) was diluted in sterile saline (0.9% w/v NaCl) to a concentration of 200 μg/mL and used immediately.

##### Immunization of animals

H-2d haplotype BALB/c mice (Charles River breeding facilities, UK) were kept within the university’s animal facilities with standard conditions of temperature and humidity, a 12 h lighting cycle and with access to food and water *ad libitum*. Groups of five BALB/c mice (6–7 weeks of age, approximate equal sizes and weights) were immunized subcutaneously with rMafA 2/3 in saline, adsorbed to Al(OH)_3_, in Zwitterion (Zw) 3–14 micelles with or without MPLA and liposomes with or without MPLA. Individual mice were immunized with 20 μg of recombinant protein on days 0, 14, and 28. Groups of five mice were also sham immunized with no protein, and another group was designated for collecting normal mouse serum (NMS). Mice were bled terminally on day 42 through cardiac puncture under ketamine anesthesia (100 µL/10 g mouse weight administered intraperitoneally per mouse), and all sera were prepared from blood and stored individually at −20°C until needed.

The services of Davids Biotechnologie GmbH (Regensburg, Germany) were used to generate polyclonal rabbit antisera to rMafA 2/3 protein. A single NZW rabbit was immunized with protein using GERBU Adjuvant Plus (GERBU Biotechnik, Heidelberg, Germany) on days 0, 14, 28 and 42, injecting 100 μg subcutaneously on each occasion. A pre-immune serum bleed was taken on day 0 before injection, and the rabbit was bled terminally by cardiac puncture under anesthesia on day 63. Processed serum was stored in aliquots at −20°C until needed.

### Analysis of immune responses

The quality and specificity of the murine and rabbit antisera to rMafA 2/3 was assessed as follows:

#### ELISA (enzyme-linked immunosorbent assay)

This was done as previously described [37]. Briefly, flat-bottomed Nunc Maxisorp microtitre plates (Thermofischer, UK) were coated overnight at 37°C with rMafA 2/3 or P9-17, FA1090 or 12CFX_T_039 (AR205) OM preparations (1 μg ml^−1^) in 0.05 M-sodium carbonate buffer, pH 9.6. Plate wells were washed three times with PBS containing 0.05% (v/v) Tween 20 (PBST) and blocked with PBST containing 1% (w/v) BSA with incubation for 1 h at 37 °C. Test sera were serially diluted in PBST-BSA and incubated for 1 h at 37°C. After washing, plate wells were incubated with horseradish peroxidase-conjugated goat anti-mouse (1/2000) and anti-rabbit (1/2000) Ig (Bio-Rad) antibody. For determining IgG1 and IgG2a antibodies, plate wells were incubated with horseradish peroxidase-conjugated goat anti-mouse IgG1 (1/2000, Invitrogen, A10551) or IgGa2 (1/2000, Invitrogen, A10685) antibodies for 1 h at 37°C. After a final wash with PBST, substrate 3,3“,5,5”-tetramethylbenzidine (Merck, UK) and H_2_O_2_ was added for 10 min and the reaction stopped with the addition of 2N H_2_SO_4_. Absorbance was measured at λ450 nm and the ELISA titer, extrapolated from the linear portion of the serum titration curve, was taken as the reciprocal of the dilution which gave an increase in absorbance of 0.1 U after 10 min.

#### SDS-PAGE and western immunoblotting

SDS-PAGE was done using a 12.5% (w/v) acrylamide gel as described previously [38]. Briefly, recombinant rMafA 2/3, OM preparations and whole-cell lysate preparations were loaded at 20 μg per well. The separated proteins were transferred to nitrocellulose paper (Schleicher and Schuell BAS, 0.45 μm, Whatman, London, UK) using a Trans-Blot Semi-Dry transfer cell (Bio-Rad) as described previously [39], with a current limit of 0.8 mA/cm^2^ for 1 hour. The nitrocellulose sheets were washed twice in Tris-buffered saline, pH 7.5, containing 0.05% (v/v) Tween-20 (TBST) and blocked in TBST containing 5% (w/v) skimmed milk powder for 1 h at 25°C. Serial dilutions of antisera were made in TBST containing 1% (w/v) glycerol and added to the strips which were then incubated for 1 h at 25 °C. After washing, the strips were reacted for 1 h at 25°C with alkaline phosphatase-conjugated goat anti-mouse/rabbit Ig (Bio-Rad) diluted 1/3000 in TBST containing 5% (w/v) skimmed milk powder. Immunological reactivity was detected with substrate solution containing nitro-bluetetrazolium and 5-bromo-4-chloro-3-indolylphosphate (Bio-Rad, Hemel Hempstead, United Kingdom) according to the manufacturer’s instructions [39]. PageRuler Pre-stained Protein Ladder (Thermo Scientific, UK, catalog number #26616, 10-170kDa) was used.

#### Flow cytometry

Flow cytometry was used to examine antibody binding to gonococci (P9-17, FA1090, AR205) following a protocol described previously [26]. Briefly, a culture of bacteria grown for ≤16 h was harvested by centrifugation, and washed twice with sterile PBS containing 1% (w/v) BSA and suspended to a concentration of ~2 × 10^8^ CFU/ml. Next, bacteria (l ml) were centrifuged (2,200 × *g* for 3 minutes), suspended in 200 μl of pooled murine/rabbit sera (tested from from 1/10 to 1/400), and incubated at 37°C for 30 min. After washing with PBS, bacteria were incubated with 100 μl of a 1/50 dilution of fluorescein isothiocyanate (FITC)-conjugated rabbit anti-mouse IgG (Dako, Agilent, Stockport, UK) or goat anti-rabbit Ig-FITC conjugate (1/100, Merck) at room temperature for 30 min. Bacteria were fixed with a 0.4% (w/v) paraformaldehyde solution at room temperature for 30 min and analyzed on a FACSAria flow cytometer. Signals were compared using FlowJo software (BD Biosciences, USA).

#### Human serum complement bactericidal assay (hSBA)

The hSBA procedure was done following the protocol of McQuillen et al. [40] with modifications as described previously [37,41]. Gonococci were grown for <16 h on GC agar plates, and the hSBA assays were done in sterile 96-well microtiter plates with lids (Greiner Bio-One, UK). Each consisted of i) 25 µl of bacteria (~1,000 Colony Forming Units, CFU) in Dulbecco’s modification of PBS (PBSB), ii) 17 µl of normal human serum (NHS) as the exogenous complement source, iii) 10 to 25 µl of serially diluted pooled test serum, and iv) PBSB containing 1% (v/v) decomplemented fetal calf serum (dFCS) to make up the final volume of 100 μL. Pre-screened Normal Human Serum (NHS) from a volunteer without a history of gonococcal or meningococcal infection and with written consent provided, was used for all hSBA experiments. Control wells included no serum or serum from sham-immunized animals, with decomplemented NHS prepared by heat inactivation at 56°C for 30 min; this was to confirm that bactericidal activity was due to a complement-mediated mechanism and that no other factors in mouse/rabbit sera were contributory. To validate the bactericidal assay, each assay included wells with anti-gonococcal P9-17 OM serum of known bactericidal activity (e.g. at the 50% endpoint serum dilution) as a positive control [26]. The plates were incubated for 1 h at 37°C with 5% (v/v) CO_2_ and 15 µl aliquots were plated in triplicate on GC agar plates, and colonies were counted 24–48 h later (ProtoCOL; Synoptics Ltd., Cambridge, United Kingdom). Bactericidal activity was determined by comparing the numbers of bacteria surviving with serum and complement to those surviving with complement but without test serum. Sera exhibiting > 50% bactericidal activity in two or more dilutions were considered positive. The 50% hSBA titers for each serum pool were determined from a minimum of *n* = 3 independent experiments and are presented as median values with the observed range within the experiments.

### Estimation of levels of MafA 2/3 among gonococcal isolates

Whole cell lysates were prepared for each of the 50 *N. gonorrhoeae* isolates belonging to the CDCP/FDA AR bank, and for strains FA1090 and P9-17. Protein content was estimated with the BCA assay, and lysates separated by SDS-PAGE and blotted. Blots were reacted with rabbit polyclonal anti-MafA 2/3 sera (1/100) and the density of each scanned immunoblot band was measured using ImageJ [42], as described previously [26,43]. The samples were run on three independent gels and in the absence of an antibody reacting with an internal gonococcal control, the 70kDa protein marker band was used as an inter-gel standard. The density readings were normalized against the average of the density readings of the 70kDa protein marker band of the gels, and data were plotted as the mean of three independent measurements of density with standard deviations.

### Effect of rabbit anti-rMafA 2/3 serum on interactions of N. gonorrhoeae with human cells in vitro

Human Chang conjunctival epithelial cells (European Type Culture Collection, Porton Down, United Kingdom) were cultured in the wells of sterile 96-well cell culture plates (Nunc) at 37°C in Dulbecco’s Modified Eagle’s Medium supplemented with Glutamax-1 and sodium pyruvate (DMEM) (Lonza, United Kingdom) and 10% (v/v) decomplemented fetal calf serum (dFCS) (Lonza). Cells were cultured in a humidified atmosphere at 37°C with 5% (v/v) CO_2_. Prior to infection, the medium was changed to remove the antibiotics. Aliquots of *N. gonorrhoeae* bacteria (10^6^ CFU in 1 mL) were mixed with rabbit anti-rMafA 2/3 serum (1% v/v) and the corresponding pre-immune serum as control (1% v/v) and incubated for 1 h at 37°C and then aliquots of 100 μL added to cells in wells in triplicate (equivalent to 10^5^ bacteria/monolayer), with incubation for 3 h at with 5% (v/v) CO_2_. Next, wells were washed with sterile phosphate buffered saline, pH 7.4 and saponin lysis buffer (100 μL/well of 1% (w/v) saponin (Merck, UK), 1% (v/v) dFCS in PBS) added for 15 min., and lysates pooled, and bacteria quantified by viable counting on GC agar plates. Controls were cells infected with bacteria alone. The percentage inhibition of bacterial association was calculated with the equation 100-(bacteria treated with serum/control bacteria) ×100.

### Statistics

GraphPad Prism 10 (GraphPad Software, Inc., San Diego, CA, USA) was used to determine statistical significance using one-way analyses of variance (ANOVA) with Tukey’s multiple comparison test. For ELISA data, one-way analysis of variance (ANOVA) with Dunnett’s multiple comparison test was used on non-transformed arithmetic data to compare mean values. Data were also analyzed using a paired sample t-Test when required (IBM-SPSS). Statistically significant *p* values were <0.05.

### Ethics declaration

The complete study was conducted between October 2021 and September 2024; the animal study was done between July 2023 and October, 2023. The research adhered to the animal experimentation regulations set forth by the United Kingdom Home Office and approved under the Animals Scientific Procedures Act, 1986 (PPL P856AD633; Developing vaccines for *Neisseria gonorrhoeae* infections). The study was authorized by the Animal Welfare and Ethics Review Board at the authors’ institution, the University of Southampton. Animal health and welfare was assessed daily by qualified animal technicians and no animals suffered significant adverse effects. Davids Biotechnologie GmbH has a permit from the Veterinäramt Regensburg for housing specific-pathogen free, healthy rabbits according to §11 TierSchG (Az31.4.4/ScP1). The company is registered for immunization of animals under the Aketenzeichen: AZ 2532.44/14 at the approving authority Umweltamt Regensburg/Veterinärwesen. All immunizations were done in accordance with National Institute of Health standards for animal welfare (NIH animal welfare number A5646-01). Permission to draw blood from adult volunteers for preparing serum complement is covered by the Hampshire A Research Ethics Committee reference approval 13/SC/0416. Informed consent with signatures for providing blood was obtained from all volunteers. The study conformed to the World Medical Association Declaration of Helsinki – Ethical Principles For Medical Research Involving Human Participants (https://www.wma.net/policies-post/wma-declaration-of-helsinki/). The study has adhered to the ARRIVE guidelines.

## Results

### Conservation and expression of MafA 2/3 in Neisseria gonorrhoeae

**Figure 1.**
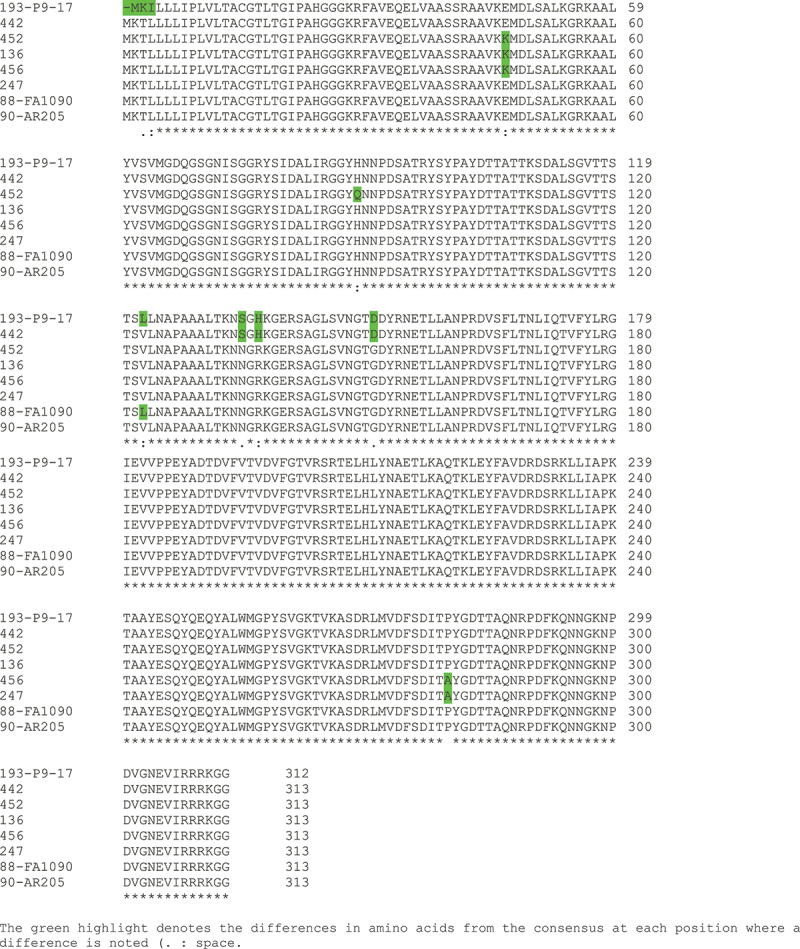
Clustal alignment of the top seven Allele encoded MafA 2/3 proteins and protein encoded by strain P9-17 (Allele 193).

**Figure 2.**
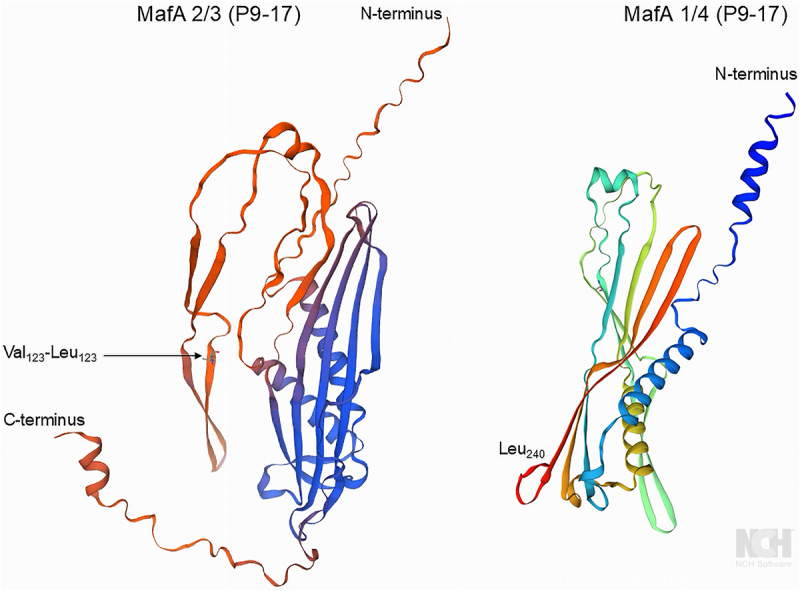
Putative structure of the MafA 2/3 and MafA 1/4 proteins derived with AlphaFold-Swiss model. In MafA 2/3 the single amino acid change at position 123 is shown, and MafA 1/4 is truncated at Leu_240_.

**Figure 3.**
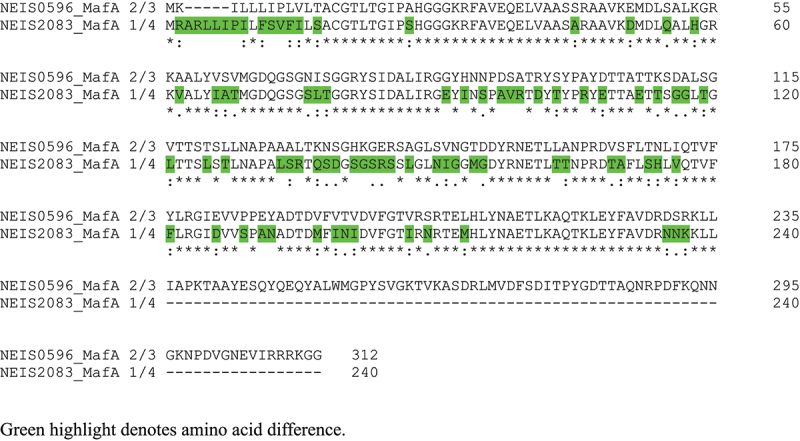
Clustal alignment of NEIS0596 MafA 2/3 with NEIS2083 MafA 1/4 from strain P9-17.

Analysis of NEIS0596 in *N. gonorrhoeae* isolates in the http://pubmlst.org/neisseria/database [34] (accessed January 2025) identified 287 alleles covering 17,947 isolates, and 13,986 isolates for which no allele was assigned (Supplementary Dataset 1). For those isolates with no allele assigned, examination of the sequence bins in PubMLST showed that they were empty, i.e. no genomes were available. Analysis of the translated proteins encoded by the nucleotide sequences of the 287 different alleles and Clustal generation of a dendrogram identified 101 non-redundant NEIS0596 allelic amino acid sequences (Supplementary Dataset 1, Supplementary Figure S1&S2). Thus, 49% (8747/17947) of *N. gonorrhoeae* isolates expressed MafA 2/3 protein encoded by Allele 90 and 26% (4598/17947) expressed protein encoded by Allele 88. Together these two alleles covered 74% of the isolates in the database, and extending to five and then to seven alleles would provide 87% and 91% coverage respectively (Supplementary Dataset 1). Clustal alignment of the top 7 allele amino acid sequences showed a high degree of conservation (Figure 1). The Allele 90 and 88 encoded MafA 2/3 proteins were identical except for one single amino acid change (Val_123_-Leu_123_) and across the top 7 allele proteins, the degree of amino acid sequence conservation was ~98% (Figure 1). We generated a predicted structure *in silico* using an AlphaFold template and showed the position of the single amino acid change between Allele 90 and 88 (Figure 2). In addition, we also compared the amino acid sequences of the MafA 2/3 and MafA 1/4 proteins with Clustal (Figure 3) and showed that MafA 2/3 has 312 amino acids (with a molecular weight of 33.8 kDa) and MafA 1/4 is truncated at amino acid 240 (with a molecular weight of 25,938 daltons). Within amino acid sequence 1–240, the proteins only shared 68% amino acid similarity (Figure 3). The putative structure of MafA 1/4 is shown in Figure 2 for comparison.

In our experimental study of the antigenicity of rMafA 2/3, we expressed the protein of our laboratory strain P9-17. On analysis through the database, we found that P9-17 expressed Allele 193 MafA 2/3 protein, and this allele accounted for only 0.04% of isolates in the database (Supplementary Dataset 1). Nevertheless, Allele 193 P9-17 MafA 2/3 protein shared 98% amino acid sequence similarity with Allele 90 and 88 MafA 2/3/proteins (Figure 1).

**Figure 4.**
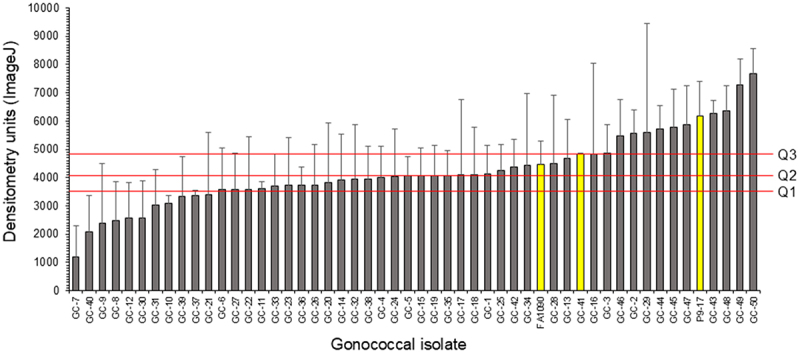
Expression of MafA 2/3 protein in a collection of gonococcal isolates quantified with image J analysis of western blots. Whole cell lysates were prepared for each of the 50 *N. gonorrhoeae* isolates belonging to the CDCP/FDA AR bank, and for strains FA1090 and P9-17. GC-41 is AR205. Blots were reacted with rabbit polyclonal anti-MafA 2/3 sera and the density of each scanned immunoblot band was measured using ImageJ. The density readings were normalized against the average of the density readings of the 70kDa protein marker band of the gels, and data were plotted as the mean of three independent measurements of density (columns) with standard deviations (error bars). Q1, Q2 and Q3 define the quartile ranges.

To investigate MafA 2/3 protein expression among different gonococcal strains, individual bacterial lysates were prepared from the CDCP/FDA AR Isolate Bank of 50 *N. gonorrhoeae* isolates. Of the 50 isolates, 27 (54%) expressed Allele 90 encoded MafA 2/3 protein and 11 (22%) expressed Allele 250 encoded protein (Supplementary Table S1), with 9 unassigned (18%). Allele 246 (2%) and 136 (2%) were expressed by single isolates. Indeed, Allele 250, Allele 90 and Allele 246 encoded proteins were identical (Supplementary Dataset 1) and therefore accounted for 78% of the total. The lysates were reacted with a polyclonal rabbit anti-rMafA 2/3 serum in three independent western blots (Supplementary Figure S3) and the level of MafA2/3 in each lysate was quantified by ImageJ densitometry (Figure 4). The median densitometry reading across the isolates was 4056 with a range from 1210 to 6463, a maximum of 7673, and quartiles Q1 = 3588, Q2 = 4056, and Q3 = 4838 (Figure 4). There were 11 isolates below Q1 (GC-7 to GC-21) and 28 isolates grouped around the median Q2 (GC-6 to GC-41), and 13 isolates in Q3 (GC-16 to GC-50). Most of the isolates expressed similar levels of MafA 2/3 except for the potential outliers GC-7 (low expressor) and GC-49 and GC-50 (high expressors).

### Production of rMafA 2/3 protein

**Figure 5.**
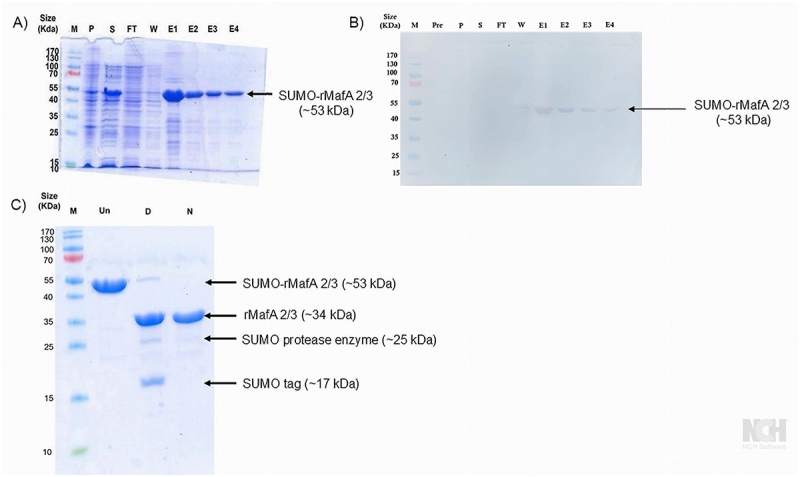
Expression and purification of rMafA 2/3 protein. a) Coomassie blue stained SDS-PAGE gel of expression of SUMO-rMafA 2/3 protein in *E. coli* Rosetta (DE3) pLysS and subsequent purification on Ni-NTA resin. B) Western blot of SUMO-rMafA 2/3 fusion protein after expression and purification. For both A) and B), M denotes *Mr* (kDa) pre-stained protein marker; pre – before IPTG induction; P, the pelleted material; S – supernatant of expressed recombinant protein; ft – unbound fraction that flowed through the column in purification step; W – wash buffer fractions (only one shown as representative); E1-E4: -consecutive elution fractions of purified SUMO-rMafA 2/3 fusion protein. C) SDS-PAGE analysis of sumo protease cleavage of recombinant SUMO-MafA 2/3 protein. Un: recombinant SUMO-MafA 2/3 protein before sumo protease digestion. D: Recombinant SUMO-MafA 2/3 protein after sumo protease digestion. N: rMafA 2/3 protein after purification and dialysis.

The mature *mafA 2/3* gene sequence (NEIS0596) from our laboratory strain P9-17 was engineered *in silico* into pMA-T plasmid for propagation in *E. coli* DH5α and initially ligated into the pET22 (+) expression vector. However, the yield of expression from the pET22(+) vector was low and insufficient for subsequent studies. Instead, we ligated next the insert into the pET-SUMO expression vector and induction of protein expression was done in LB broth with IPTG, and expressed protein was purified using Ni-NTA metal-affinity chromatography under native conditions to yield soluble SUMO-tagged recombinant rMafA 2/3 (Figure 5(A,B)). The SUMO tag was subsequently removed with SUMO protease enzyme to yield mature protein of *Mr* ~34kDa (Figure 5(C)). The yield of purified rMafA 2/3 was ~11.52 mg per liter of culture, which was sufficient for subsequent *in vivo and in vitro* studies. The SUMO tag typically appeared with Mr of 15–17 kDa on SDS-PAGE [44]. SUMO protease enzyme appeared with molecular weight ~25 kDa. The result showed visually that cleavage is more than 90% efficient in removing SUMO tag from recombinant SUMO-MafA protein.

### Antigenicity of rMafA 2/3 protein

**Figure 6.**
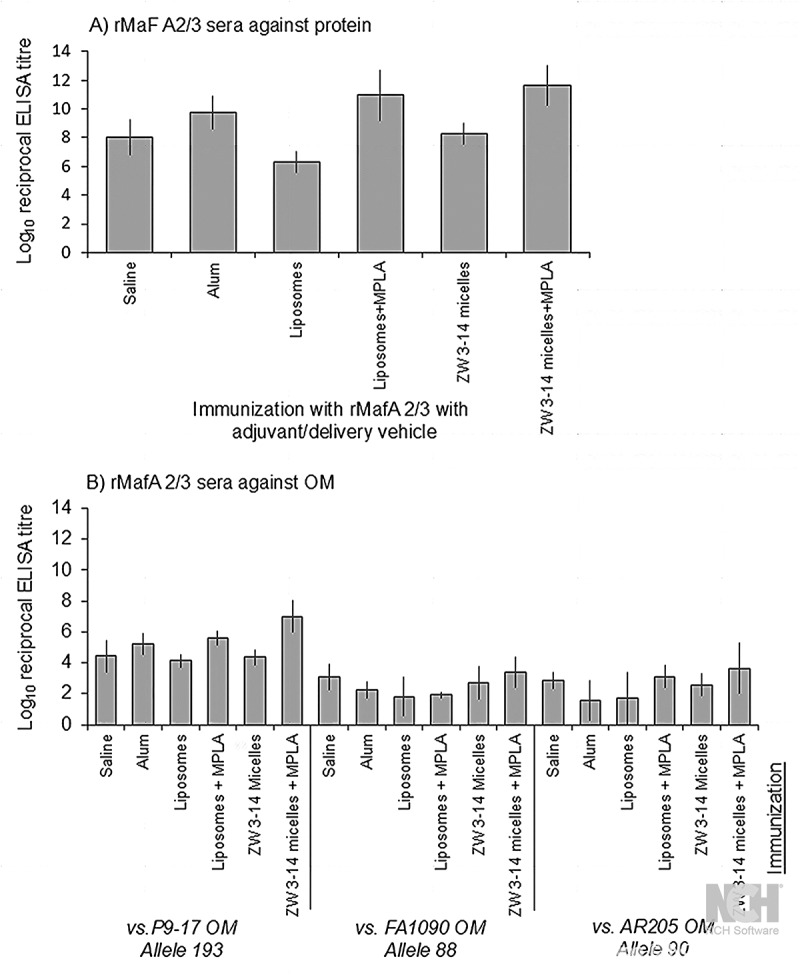
Reactivity of murine antisera raised to rMafA 2/3 protein in ELISA. A) Reactivity of antisera against recombinant protein. B) Reactivity of antisera against homologous P9-17 OM and heterologous FA1090 OM and 12CFX_T_039 (AR205) OM. The columns represent the geometric mean reciprocal ELISA titers and the error bars the 95% confidence limits, for *n* = 5 sera per group. All control sera, i.e. raised to saline, alum, liposomes ±MPLA and Zw 3–14±MPLA, as well as normal mouse sera, did not react with any of the OM preparations (titers all <1/100).

The recombinant P9-17 rMafA 2/3 protein was used to immunize mice with a variety of adjuvants and delivery vehicles, and sera were tested initially in ELISA against the homologous protein and OM preparations. Mice were chosen for immunization as an animal model for producing polyclonal sera using a statistically significant number to enable analysis of individual immune responses. Against the homologous protein, sera raised to rMafA 2/3 in saline alone had a high Geometric Mean Titre (GMT) of 108x10^6^ (Figure 6(A)), which was surpassed by adsorption to alum (GMT ~5500x10^6^). The lowest GMT was shown by sera to protein in liposomes alone (2x10^6^), but the addition of MPLA significantly increased the GMT to ~9x10^10^ (Figure 6(A)). Similarly, the addition of MPLA to the Zw 3–14 micelles increased the GMT from 202x10^6^ to ~4x10^11^ in ELISA. rMafA 2/3 sera reacted strongly with homologous P9-17 OM, with GMT for antisera raised to protein in saline and alum of ~0.3x10^5^ and ~2x10^5^ respectively. Statistically, reactivity of anti-rMafA 2/3 on homologous protein was higher than on P9-17 OM (*p* = 0.008). Addition of MPLA significantly increased the reactivity of antisera with homologous P9-17 OM; thus, for liposomes, the presence of MPLA increased the GMT from ~1x10^4^ to ~4x10^5^, and for Zw 3–14 micelles from ~2x10^4^ to ~1x10^7^ (Figure 6(B)). Statistically, the reactivity of antisera against P9-17 OM was generally higher than that observed against the heterologous OM from FA1090 and 12CFX_T_039 (AR205) (*p* = 0.003). There was no difference in serum reactivity between the heterologous OM preparations themselves (*p* > 0.4) (Supplementary Table 2). All control sera, i.e raised to saline, alum, liposomes ±MPLA and Zw 3–14±MPLA, as well as normal mouse sera, did not react with rMafA 2/3 protein or any of the OM preparations (titers all <1/100).

**Figure 7.**
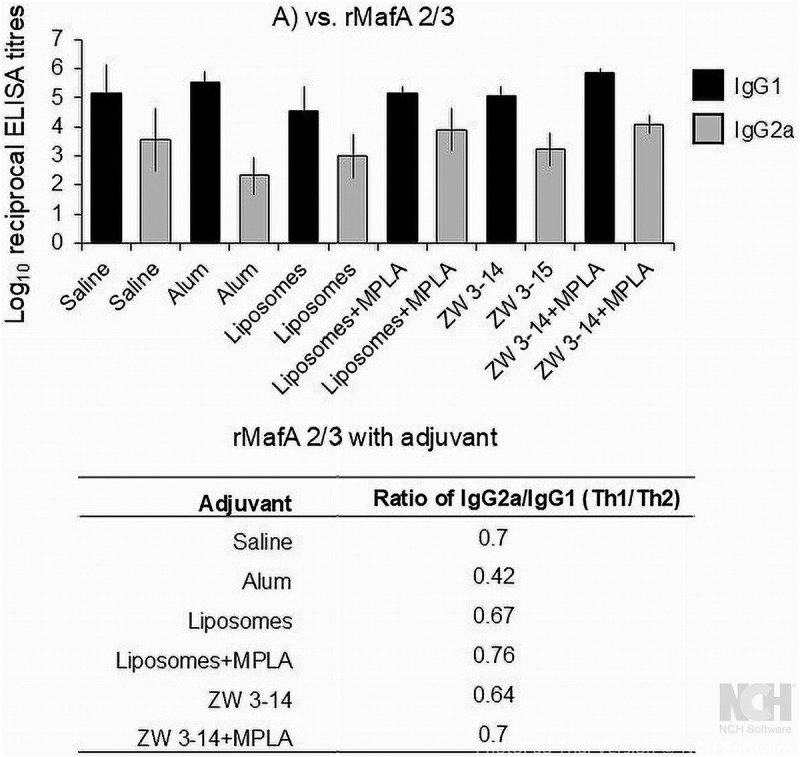
Measurement of anti-rMafA 2/3 IgG1 and IgG2a subclass antibodies in murine antisera. The columns represent the geometric mean reciprocal IgG1 and IgG2a ELISA titers and the error bars the 95% confidence limits, for *n* = 5 sera per group. All control sera, i.e raised to saline, alum, liposomes ±MPLA and Zw 3–14±MPLA, as well as normal mouse sera, did not react with any of the OM preparations (titers all <1/100). The table defines the IgG2a/IgG1 ratios of the log titer values.

The distribution of IgG1 and IgG2a antibodies in mouse serum raised to rMafA 2/3 with the different adjuvants was examined also by ELISA on rMafA 2/3 as antigen (Figure 7). In general, the murine response to rMafA 2/3 with the adjuvants and delivery vehicles was dominated by the IgG1 antibody isotype. Statistically, there were no significant differences in the IgG1 levels induced using saline, alum, liposomes ± MPLA and Zw-314 alone, although the mean GMT levels induced with rMafA 2/3 in Zw 3–14 + MPLA were higher (Supplementary Table 2). Lower levels of IgG2a antibodies were induced with rMafA 2/3 in saline (GMT ~4000), alum (GMT ~200), liposomes (GMT ~1000) and Zw 3–14 micelles (~2000) alone. The addition of MPLA to liposomes and the Zw 3–14 micelles increased the IgG2a GMT titers to ~ 8000 and ~13,000, respectively (Figure 7), but these data were not statistically significant (*p* > 0.2; Supplementary Table 2). The levels of IgG1 induced with each of the adjuvants were significantly higher than the levels of IgG2a correspondingly induced (*p* <0.05; Supplementary Table 2). The log IgG2a/IgG1 ratio for sera raised with alum was 0.42, whereas for saline and all the other adjuvants it ranged from 0.64 to 0.76.

**Figure 8.**
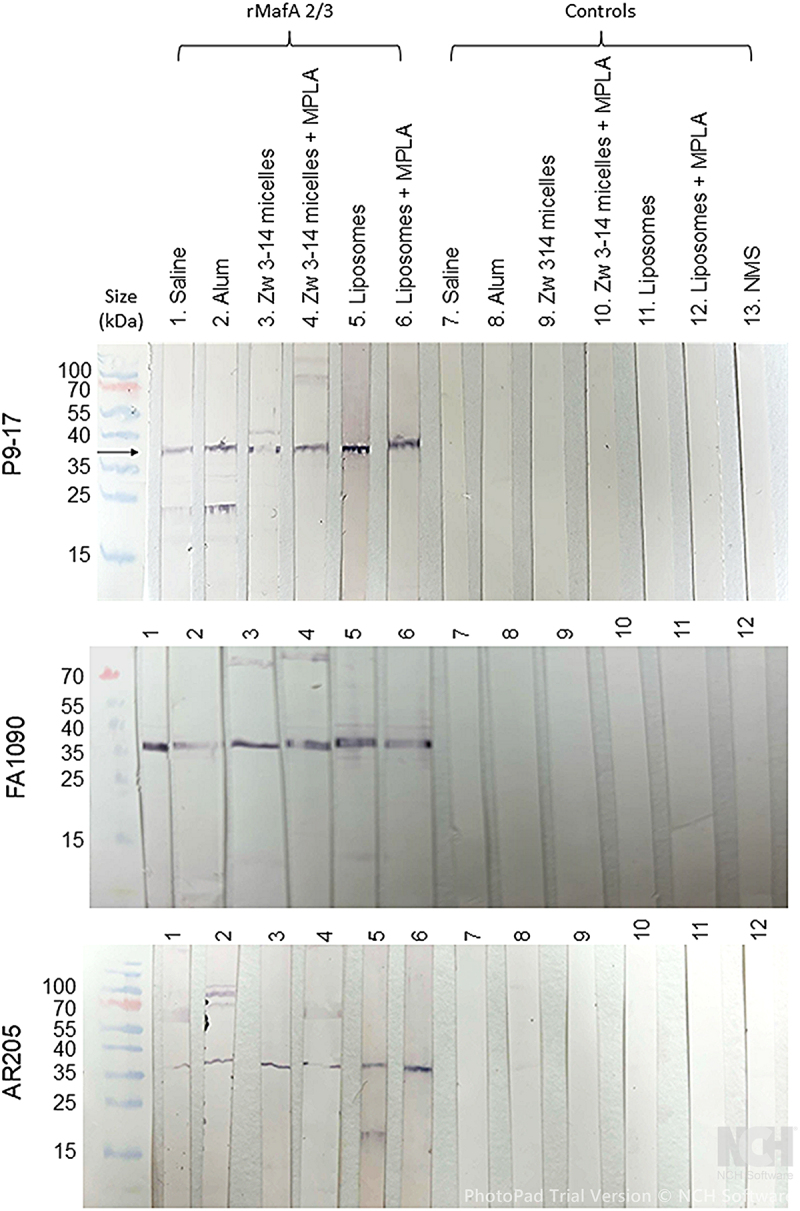
Reactivity of murine antisera with gonococcal outer membranes containing MafA 2/3 in western blot. Pooled antisera to rMafA 2/3 in saline and with a variety of adjuvants were reacted with P9-17, FA1090 and AR205 OM preparations separated by SDS-PAGE and blotted onto nitrocellulose. The arrows denote the position of the reactive om band with anti-rMafA 2/3sera, and the absence of a reactive band with control sera. Images are representative of western blots done *n* = 3 times.

**Figure 9.**
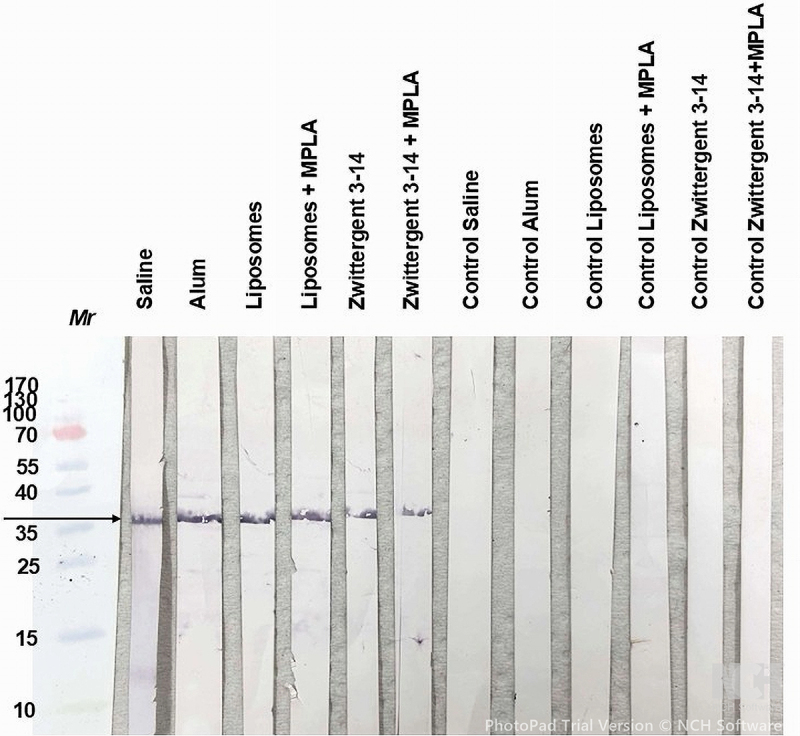
Reactivity of murine antisera with the rMafA 2/3 protein in western blot. Pooled antisera to rMafA 2/3 in saline and with a variety of adjuvants were reacted with the rMafA 2/3 protein in western blot. A single band of reactivity (denoted by the arrow) is observed with anti-rMafA 2/3 sera, and the absence of a reactive band with control sera. Images are representative of western blots done *n* = 2 times.

In western blot experiments, antisera to rMaFA 2/3 protein in saline and all the adjuvant mixtures and delivery vehicles recognized a single band of *Mr* slightly higher than the predicted *Mr* of 34 kDa, which could be the result of a post-translational modification *in vivo* (Figure 8). All the antisera groups cross-reacted with both strain FA1090 and AR205 OM (Figure 8). In some instances, we observed the presence of minor reactive bands, in particular a band of ~25kDa in sera from mice immunized with alum-adjuvanted rMafA 2/3 against P9-17 OM; speculatively this serum pool could be recognizing the MafA 1/4 of *Mr* 26 kDa. Minor bands of ~ 70 kDa were also observed in sera from mice immunized with alum or Zw 3–14 micelles with and without MPLA against the OM preparations, and speculatively these could represent dimers. No reactivity was observed with sera from animals receiving sham immunizations or with normal mouse serum (Figure 8). As positive control, sera were also tested against the rMafA 2/3 protein in western blots; positive reactivity was seen against a single band of Mr slightly higher than the predicted Mr of 34 kDa (Figure 9), identical to the reactivity observed for sera tested against MafA 2/3 in OM preparations (Figure 8).

**Figure 10.**
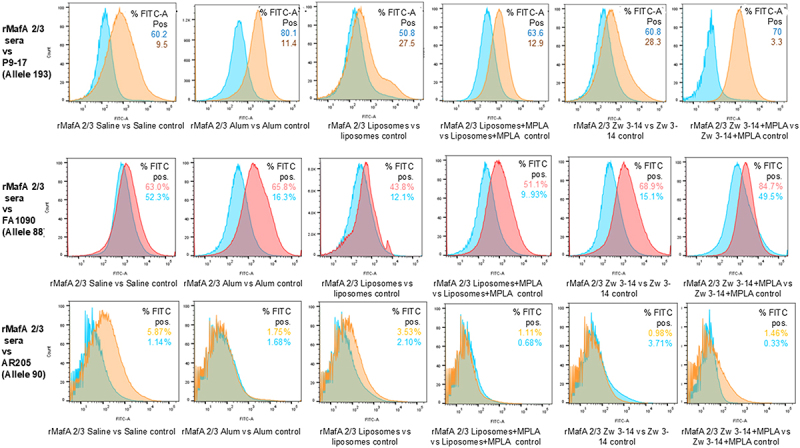
Reactivity of murine antisera raised to rMafA 2/3 with whole bacteria examined with flow cytometry. Pooled antisera to rMafA 2/3 and different adjuvants were incubated with gonococcal strains P9-17, FA1090 and AR205 and then FITC-conjugated rabbit anti-mouse IgG antibody. Control was pooled serum from mice immunized with corresponding adjuvant alone. Fixed samples were analyzed on a FACSAria flow and % FITC-positive staining is shown for each graph. Data were representative of *n* = 2 experiments.

The ability of antisera raised to rMafA 2/3 to recognize native MafA 2/3 protein on the surface of gonococci was assessed by flow cytometry. Sera raised to rMafA 2/3 showed high levels of percentage FITC positive surface labeling of homologous strain P9-17 (Figure 10). For example, fold increases with sera raised to rMafA 2/3 in saline or alum was ~6–7. When using liposomes or Zw 3–14 alone, the increases were ~2-fold, but these increased significantly when MPLA adjuvant was incorporated, rising to 5- and 21-fold respectively (Figure 10). There was also cross-reactivity of some of the sera with the heterologous FA1090 strain, e.g. sera raised with alum, liposomes ±MPLA and Zw 3–14 showed ~4–5-fold increases in percentage FITC positive surface labeling. When comparing between the groups of sera, there were no significant differences in percentage FITC positive surface labeling between P9-17 and FA1090 bacteria (*p* = 0.393). By contrast, the reactivity of serum groups against both P9-17 and FA1090 was significantly higher (*p* <0.001) than that observed against the other heterologous AR205 strain (Figure 10), which showed no significant fold-increases compared to the controls.

### Serum bactericidal activity (SBA)

The ability of rMafA 2/3 to produce functional antibodies was assessed using a human serum complement bactericidal assay. Initially, murine antisera were tested against homologous strain P9-17 (Allele 193) (Table 1). Weak bactericidal activity toward P9-17 was generated using saline, alum, and liposomes (median 50% SBA values of 4–8) (Table 1). However, the addition of MPLA to the liposomes increased the SBA titers to 256. In addition, median 50% SBA titers of 256 were recorded with sera raised to rMafA 2/3 with Zw 3–14, which was not increased after the addition of MPLA (Table 1). Bactericidal activity was observed against the heterologous strain FA1090, with titers ranging from 4 to 16 with all the adjuvants, except for sera raised to rMafA 2/3 in Zw 3–14+MPLA, which demonstrated a median 50% SBA titer of 64 (Table 1). No bactericidal activity was observed for any of the murine sera when tested against heterologous strain AR205 (Table 1).

**Table 1.** Bactericidal activity of murine antisera and the rabbit antiserum raised to rMafA 2/3 protein.

			Median reciprocal 50% bactericidal titer against strain			
Animal	Antigen	Adjuvant		P9-17 (Allele 193)	FA1090 (Allele 88)	12CFX_T_039, AR205 (Allele 90)
Mouse	rMafA 2/3	Saline		4	16	<4
		Alum		4	16	<4
		Liposomes		4	4	<4
		Liposomes ± MPLA		256	4–8	<4
		Zw 3–14 micelles		256	4	<4
		Zw 3–14 micelles ± MPLA		256	64	<4
Mouse	No antigen control	Saline		<4	<4	<4
		Alum		<4	<4	<4
		Liposomes		<4	<4	<4
		Liposomes ± MPLA		<4	<4	<4
		Zw 3–14 micelles		<4	<4	<4
		Zw 3–14 micelles ± MPLA		<4	<4	<4
Rabbit	rMaFA2/3	Gerbu		2048	2048	2048–4096

Data shown are the median values for bactericidal assays done at least 3–5 independent times for each antiserum pool. Normal mouse serum and rabbit pre-immune serum showed titers of <4.

### Preliminary study – can antisera to rMafA 2/3 inhibit the association of gonococci to human cells cultured in vitro?

**Figure 11.**
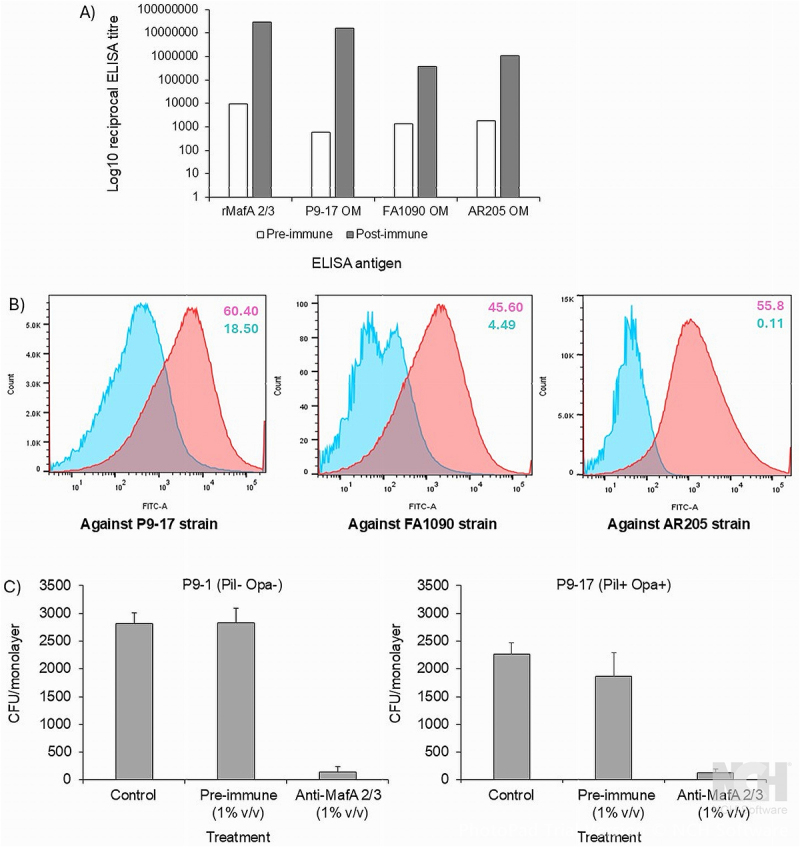
A) reactivity in ELISA of rabbit pre- and post-immune serum raised to rMafA 2/3 protein against homologous protein and OM preparations. B) Flow cytometry analysis of rabbit antiserum reactivity with P9-17, FA0190 and AR205 isolates. Data are representative of *n* = 2 independent experiments. The pink shading represents the rabbit post-immune serum and the blue shading the pre-immune serum. C) Anti-MafA 2/3 serum inhibition of gonococcal association with human Chang conjunctival epithelial cells. P9-1 and P9-17 bacteria (10^6^ colony forming units) were incubated with 1% (v/v) decomplemented rabbit pre-immune serum and 1% (v/v) anti-rMafA 2/3 serum for 1 h, and aliquots then added to Chang cells in triplicate wells, followed by incubation for 3 h. Adherent bacteria were quantified by viable counting. The columns represent the means and the bars the standard errors of the means of *n* = 3 independent experiments.

As the murine antisera were limiting, we also produced in the current study, anti-rMafA 2/3 serum in one rabbit. This provided a larger volume of serum that enabled us to i) analyze the MafA 2/3 expression levels by western blot described above, ii) examine the effect of sialylation of the bacteria on bactericidal activity, and iii) do *in vitro* cell culture inhibition of adhesion experiments. Anti-rMafA 2/3 rabbit serum reacted strongly with recombinant protein and OM preparations from P9-17, FA1090 and AR205 in ELISA (Figure 11A). The pre-immune serum had titers of ~9000, 600, 1300 and 1800 against rMafA 2/3, P9-17, FA1090, and AR205 OM, respectively, and the corresponding post-immune titers were ~29x10^6^, 2x10^6^, 0.04x10^6^ and 0.11 x10^6^, respectively. In flow cytometry (Figure 11B), significant shifts were observed with the post-immune rabbit serum against P9-17, FA1090 and AR205 strains. The serum also recognized MafA 2/3 in western blots (Supplementary Figure S3). This serum recorded a pre-immune 50% SBA titer of 4 and a post-immune titer of 2048 against non-sialylated exemplar strain P9-17 (Table 1), and when tested against sialylated P9-17, the bactericidal activity of post-immune sera was reduced to 64. In addition, the post-immune rabbit serum killed strain FA1090, with a mean 50% SBA titer of 2048, and strain AR205 with a mean 50% SBA titer of 2048–4096 (Table 1).

In preliminary experiments, we used this high-volume rabbit anti-MafA 2/3 serum to test the hypothesis that antibodies to the protein could inhibit gonococcal association with human cells *in vitro*. In these experiments, we used Chang conjunctival epithelial cells, which have been used extensively to study host cell-gonococcal interactions such as Opa-mediated invasion, proteoglycan interactions and intracellular signaling [45–49]. In our study, we infected Chang cells with Pil^+^ Opa_b_^+^ and Pil^−^ Opa^−^ variants, to examine whether the presence of the major adhesin/invasins impacted on the ability of anti-MafA 2/3 antibodies to inhibit association, e.g. through any potential steric interference of antibody binding. When preincubated with gonococcal strains P9-1 (Pil^−^ Opa^−^) and P9-17 (Pil^+^ Opa_b_^+^), the anti-MafA 2/3 sera reduced bacterial association by ~95% compared to the corresponding pre-immune serum (*p* <0.05) (Figure 11C), in cultures that had been infected for 3 h.

## Discussion

In this study, we produced a soluble recombinant MafA 2/3 protein of our laboratory strain P9-17 using the pHYRSF53-SUMO system and used it to immunize mice with a variety of different adjuvants and delivery vehicles. The principal findings were that i) immunization with rMafA 2/3 protein induced antibodies that were bactericidal for gonococci, ii) the most effective responses were induced with protein incorporated into liposomes or Zwittergent micelles that contained the adjuvant MPLA, iii) functional activity correlated with serum reactivity against the homologous protein and OM in ELISA, and recognition of the protein in OM western blots and on the bacterial surface using flow cytometry, and iv) anti-MafA 2/3 sera inhibited gonococcal association with human cells cultured *in vitro*. Functional bactericidal antibodies could be induced by immunization with protein in saline alone, which we have previously observed with another adhesin, Ng-ACP [26]. However, the most effective bactericidal responses were with formulations that include the Toll-*Like* Receptor 4 agonist MPLA. Liposomes containing MPLA are licensed for human vaccines [50], whereas the Zwittergent 3–14 detergent is not licensed for human use because of toxicity issues, despite its excellent adjuvant properties [51]. The increase in bactericidal activity generated by the addition of MPLA may be due to the adjuvant’s ability to induce a broader spectrum of antibody subclasses, including those most effective in complement activation [36].

In our current study, the highest murine bactericidal titers against the homologous strain were 256, and MafA 2/3 protein joins the library of individual gonococcal antigens that are stably expressed and highly conserved that have been reported to induce serum bactericidal activity *in vitro* [10]. However, it is difficult to make direct comparisons between different studies, due to the use of different gonococcal strains and the absence of a standardized hSBA [52]. SBA is an accepted correlate of protection for licensing meningococcal vaccines, but the role of vaccine-induced anti-gonococcal antibodies in preventing and/or clearing mucosal infection is unclear. SBA is a plausible surrogate of protection, in that it potentially measures functional immunity, i.e. it can discriminate between killing of gonococci and simple antibody binding to the bacterium. The assay is a useful tool for early evaluation of experimental gonococcal vaccines, but its predictive value in humans requires demonstration. Regardless, immunization of mice with recombinant α-BamA (NGO1801), NGO2054, MetQ (NGO2139), TamA (NGO1956), and LptD (NGO1715) antigens, which were also identified by proteomics-bioinformatics approaches, induced bactericidal titers ranging from 16 to 512 against different strains [53,54]. Several other proteins capable of inducing bactericidal antibody include proteins involved in colonization and invasion (PilQ_406–770_, titers of 400 to 800) [55]; cell division (NGO1549, FtsN; cell divisome protein and NGO0265, predicted cell division protein) [56]; nutrient acquisition (transferrin-binding proteins, titers of 50 to 100) [57]; immune evasion (porin peptides, titers of ~320) [37]; NspA (titers of ~100 to 1,000) [58]; LOS 2C7 mimotopes (with percentages of killings calculated per microgram of IgG) [59]; a trivalent vaccine containing Adhesion and Penetration Protein (APP), NHBA and MetQ (titers from 100–800) [60]; MetQ-NHBA fusions (titers 200–1600) [61]; APP (titers 20–80) [62]; a mixture of NGO0690, NGO0948 and NGO1701 (titers ~80) [63]; MtrE (titers ~100–1000) [64]; and NHBA (titers 100–6400) [65]. Meningococcal outer membrane vaccines (OMV) have been reported to induce murine and rabbit antibodies that killed gonococci, though a direct comparison cannot be made, as these OMV are complex antigen mixtures [66,67]. However, on 10 November 2023, the UK’s Joint Committee on Vaccination and Immunization (JCVI) recommended starting a targeted 4CMenB (Bexsero) vaccination program against gonorrhea for individuals at higher risk, notably men-having-sex with men, and other groups such as trans women/gender-diverse individuals assigned male at birth, heterosexuals of similar risk and sex workers with condomless sex (JCVI advice on the use of meningococcal B vaccination for the prevention of gonorrhea – GOV.UK). Implementation began from 1 August , alongside mpox, hepatitis A/B, and HPV vaccines, where appropriate. Modeling has suggested that efficacy of the vaccine could be between 30–47%, although there are significant caveats with its use, and it will be interesting to see how adaptable the gonococcus becomes to immune pressure from this vaccine. In developing refined subunit vaccines, it is unlikely that rMafA 2/3 or any other singular antigen would be used to produce a sole gonococcal vaccine, and combinations of the best-performing antigens would likely produce a multi-component vaccine for preventing gonococcal infection [68].

The generation of bactericidal antibody in the mouse to *Neisseria* antigens is dependent on several factors, including the amount, epitope specificity, and high avidity of the antibodies induced, and on the class and ratio of complement-activating to non-complement-activating antibodies [69], all of which can be influenced by the choice of adjuvant. Where bactericidal activity is complement-dependent, murine IgG2a, IgG2b, IgG3, and IgM isotype antibodies are effective activators of complement, whereas IgA and IgGl show little or no activity [70,71]. The choice of adjuvant can bias the Th1/Th2 immune response, with Th1 responses favored by increased IgG2a and lowered IgG1, and Th2 by increased IgG1 and lowered IgG2a. For example, alum biases the murine response to Th2 [72,73], whereas MPLA or a similar glucopyranosyl lipid adjuvant (GLA) biases the response toward Th1 [74,75]. In our study, we found that the addition of MPLA to both liposomes and the Zw 3–14 micelles increased the amount of IgG2a subclass antibody, which may have contributed to the increased SBA against gonococci, compared with the other adjuvants used. This accords with the observation that IgG2a is a strong activator of complement in the mouse for SBA against gonococci [76] and meningococci [69]. However, the Th1/Th2 ratios suggested that the overall response to rMafA 2/3 in the BALB/c mouse with all adjuvants trended toward Th2 (ratio ~0.7), suggesting that subclass alone is not the only attribute important for SBA. This was clear from the observation that high SBA against gonococci was observed with sera raised to rMafA 2/3 in Zw 3–14 micelles alone, suggesting that other factors such as correct conformational folding and epitope structure, and the presentation of proteins and epitopes, are important for inducing functional antibodies [26,77,78].

We also examined whether the bactericidal activity of anti-MafA 2/3 sera were affected by sialylation of the bacteria with CMP-NANA. The gonococcus gains a survival advantage by sialylating its LOS with CMP-NANA that it collects from the urogenital tract, since this makes gonococci resistant to killing by complement found in genital secretions and serum [79]. Gonococci freshly isolated from the genital tract are resistant to serum and subculture of the bacteria in the laboratory leads to a rapid loss in resistance [29]. Treating sensitive bacteria with exogenous CMP-NANA *in vitro* can restore resistance. In the context of vaccine development, LOS sialylation has been reported to prevent complement-dependent killing by immune sera generated in response to different OM antigens such as porin and porin peptides [80–82], Ng-ACP [26], and with the 2C7 monoclonal antibody immunotherapeutic [83]. In the current study, we found that sialylation of gonococci reduced the bactericidal effects of a high-titer rabbit anti-rMafA 2/3 serum, from 50% SBA titer of 2048 down to 64, a 32-fold reduction. However, it is still uncertain whether reduction of bactericidal activity against sialylated bacteria would disqualify rMafA 2/3 or other candidate antigens for inclusion in gonococcal vaccines. There is still merit in generating antibodies with bactericidal activity against unsialylated gonococci, based on biological observations during infection. *In vivo*, excessive LOS sialylation may block invasion of gonococci into mucosal epithelial cells but protect the organism from natural host immunity, whereas a loss of sialic acid modification conversely makes the gonococcus more invasive but more susceptible to eradication by host immune mechanisms [1,84]. In addition, gonococci with sialylated LOS were less infective than gonococci with nonsialylated LOS in the human male challenge model [85]. Cervical secretions from women with gonorrhea also contained sialidase enzymes that could remove sialic acid from sialylated LOS, thus making gonococci potentially susceptible to bactericidal antibodies that would normally not kill sialylated bacteria [86]. Thus, one approach to inducing complement-mediated killing may involve focusing the immune response onto surface-exposed OM antigenic epitopes that are the least susceptible to the potential inhibitory effects of LOS sialylation [81].

Analysis of MafA 2/3 (NEIS0596) among *N. gonorrhoeae* isolates in the http://pubmlst.org/neisseria/database identified 101 non-redundant allelic amino acid sequences, with Allele 90 and 88 encoded proteins expressed by 49% and 26% (74% total) of the isolates, respectively. Allele 90 and 88 encoded MafA 2/3 proteins were identical except for one single amino acid change, and moreover the top 7 allele proteins showed ~98% amino acid sequence. In addition, all 50 of the CDC/FDA isolates expressed the protein. Thus, MafA 2/3 appeared to be a highly conserved and expressed gonococcal protein, and coupling the limited variability with the potential to induce cross-protective responses suggests that a broadly functional vaccine may only need to contain Allele 90 and/or 88 proteins. The potential to induce cross-functional antibody responses was shown by the observation that murine antibodies raised to Allele 193 rMafA 2/3 (laboratory strain P9-17) was able to kill strain FA1090 (Allele 88), albeit with reduced SBA titers. Allele 193 and Allele 88 proteins were 98% identical, which could account for the cross-reactivity. However, no killing was observed against strain AR205 that expressed Allele 90 MafA 2/3: it is possible that the single amino acid change between Allele 88 and 90 proteins is sufficient to negate the murine bactericidal activity, or our Allele 88 strain FA1090 is more sensitive to complement-mediated killing than Allele 90 AR205. Furthermore, we noted that murine polyclonal antisera reacted with AR205 on western blot and ELISA, but did not react with whole bacteria in flow cytometry, suggesting that binding of antibodies to this heterologous strain may depend possibly on conformation. Further studies would involve producing the dominant Allele 90 and 88 rMafA 2/3 proteins and examining their potential to induce bactericidal activity against a larger range of gonococcal isolates expressing other allelic proteins.

It is theoretically possible that antibodies generated to MafA 2/3 would recognize the MafA 1/4 protein, if they could react with shared epitopes. However, in our western blots of the 50 gonococcal isolates, the rabbit polyclonal anti-MafA 2/3 serum recognized only a single band consistent with the MafA 2/3 protein (*Mr* ~34/35 kDa) and did not recognize a lower *Mr* band of ~ 28 kDa. Thus, either these isolates did not express MafA 1/4, or the dissimilarities between MafA 2/3 and MafA 1/4 in amino acid sequence, structure, and a possible lack of shared epitopes, suggested that antibodies to the former did not recognize epitopes on the latter.

We also demonstrated in preliminary studies that antiserum raised to rMafA 2/3 in a rabbit inhibited the association of gonococci to human Chang conjunctival cells *in vitro*. The rabbit was immunized with Gerbu, an adjuvant that contains muramyl glycopeptides and solid ultra-filtrable nanoparticles of slowly biodegradable lipids with immune-potentiating, biocompatible emulsifiers and carefully adjusted synergistic and stabilizing medium (*sic*). Notably, this rabbit antiserum showed significant binding to P9-17, FA1090 and AR205 strains in flow cytometry, which correlated with significantly high SBA titers against all three strains. This contrasts with the activity of murine anti-rMafA 2/3 sera against AR205 strain in flow cytometry, where no significant reactivity and no bactericidal activity was observed. Indeed, the rabbit SBA titers were significantly higher than those induced in the mouse and suggest that the choice of animal model may be important in assessing the immunogenicity of gonococcal vaccine antigens. It is possible that the higher reactivity with rabbit serum over murine sera is due to better antibody affinity and specificity and higher antibody concentration/mL, although this conclusion remains speculative as different adjuvants were used.

The ability of a vaccine containing rMafA 2/3 to induce antibodies that inhibit association suggests that such antibodies may interfere with gonococcal adherence/colonization *in vivo* and this would be an important attribute complementary to bactericidal activity. Furthermore, the observation that neither pilus nor Opa protein expression impacted negatively on the ability of rabbit anti-MafA 2/3 serum to inhibit association, suggested that MafA 2/3 may play a role in the adherence process. In our study, there was no significant difference in the binding of piliated, Opa-expressing bacteria and non-piliated, non-Opa expressing bacteria to Chang cells, and this is probably because a short contact period of only 3 h was used to test the ability of anti-MafA 2/3 sera to inhibit association. Our preliminary experiments confirmed the hypothesis that anti-MafA 2/3 antibodies could inhibit gonococcal association and implied indirectly that MafA 2/3 could be a potential adhesin. To prove this conclusively is outside the scope of the current study and the subject of a future independent study, which could i) use primary non-transformed epithelial cells derived from the cervix, ii) compare the interactions of MafA 2/3 mutant and parent strains with cell lines *in vitro*, iii) examine whether antisera could inhibit the association of sialylated bacteria, and iv) use the mouse gonorrhea model to examine gonococcal fitness.

In summary, immunization of mice with a recombinant MafA 2/3 OM protein in liposomal and micellar formulations containing MPLA, induced antibodies that could kill gonococci *in vitro*. rMafA 2/3 should be considered as another promising OMP for further study as a component of future subunit gonococcal vaccines.

## Supplementary Material

Supplementary_Table1_revised.docx

Supplementary_Figure_3.tif

Supplementary_Table2_revised.docx

Supplementary_Figure_1.docx

Supplementary_Dataset1.xlsx

Supplementary_Figure_2.tif

## Data Availability

The authors confirm that the data supporting the findings of this study are all available within the article and its supplementary materials and raw data to accompany the article are live, as per the University of Southampton’s policies on data sharing at https://doi.org/10.5258/SOTON/D3458
