# Supplementary material for: Antigenicity in mice of a recombinant Neisseria gonorrhoeae MafA 2/3 protein
Source: Virulence. 2025 Oct 29;16(1):2580086. doi: 10.1080/21505594.2025.2580086 (PMC12574560; doi:10.1080/21505594.2025.2580086)
Supplement: Supplementary_Table1_revised.docx [file KVIR_A_2580086_SM4704.docx]

**Supplementary Table 1. MafA 2/3 alleles determined for isolates in the CDC/FDA AR Gonococcal Isolate Bank.** The sequence accession number ([https://www.cdc.gov/drugresistance/resistance- bank/currently-available.html](https://www.cdc.gov/drugresistance/resistance-%20bank/currently-available.html)) for each isolate is linked to the SRA webpage (<https://www.ncbi.nlm.nih.gov/sra/?term=SAMEA3165293>) that provides the run accession number that can be used to search the Sanger repository (<http://www.sanger.ac.uk/resources/downloads/bacteria/neisseria.html#project_3416>) that can identify the strain information. The strain information is then used to search the pubmlst/*Neisseria* webpage (<https://pubmlst.org/bigsdb?db=pubmlst_neisseria_isolates&page=query>) to identify the ID number, amino acid sequence, and encoding allele.

| **AR-Bank no.** | **GC no.** | **Sanger run accession no.** | **Sanger strain no.** | **Pubmlst/*Neisseria* ID** | **MafA2/3 allele** |
| --- | --- | --- | --- | --- | --- |
| 165 | GC-01 | [ERR854938](https://www.ncbi.nlm.nih.gov/sra/ERR854938) | 12CFX_T_043 | 37079 | 90 |
| 166 | GC-02 | [ERR854921](https://www.ncbi.nlm.nih.gov/sra/ERR854921) | 12CFX_T_026 | 37062 | 250 |
| 167 | GC-03 | [ERR855352](https://www.ncbi.nlm.nih.gov/sra/ERR855352) | 12AZI_T_002 | 37057 | 90 |
| 168 | GC-04 | [ERR854924](https://www.ncbi.nlm.nih.gov/sra/ERR854924) | 12CFX_T_029 | 37065 | 250 |
| 169 | GC-05 | [ERR854922](https://www.ncbi.nlm.nih.gov/sra/ERR854922) | 12CFX_T_027 | 37063 | 90 |
| 170 | GC-06 | [ERR854897](https://www.ncbi.nlm.nih.gov/sra/ERR854897) | 12CFX_T_001 | 37037 | 250 |
| 171 | GC-07 | [ERR854937](https://www.ncbi.nlm.nih.gov/sra/ERR854937) | 12CFX_T_042 | 37078 | 90 |
| 172 | GC-08 | [ERR854906](https://www.ncbi.nlm.nih.gov/sra/ERR854906) | 12CFX_T_010 | 37046 | N |
| 173 | GC-09 | [ERR854907](https://www.ncbi.nlm.nih.gov/sra/ERR854907) | 12CFX_T_011 | 37047 | 90 |
| 174 | GC-10 | [ERR854900](https://www.ncbi.nlm.nih.gov/sra/ERR854900) | 12CFX_T_004 | 37040 | 250 |
| 175 | GC-11 | [ERR855365](https://www.ncbi.nlm.nih.gov/sra/ERR855365) | 12AZI_T_015 | 37520 | N |
| 176 | GC-12 | [ERR854869](https://www.ncbi.nlm.nih.gov/sra/ERR854869) | 12CFX_T_045 | 37081 | 250 |
| 177 | GC-13 | [ERR855355](https://www.ncbi.nlm.nih.gov/sra/ERR855355) | 12AZI_T_005 | 37510 | N |
| 178 | GC-14 | [ERR855325](https://www.ncbi.nlm.nih.gov/sra/ERR855325) | 12AZI_C_010 | 37480 | 90 |
| 179 | GC-15 | [ERR855356](https://www.ncbi.nlm.nih.gov/sra/ERR855356) | 12AZI_T_006 | 37511 | 136 |
| 180 | GC-16 | [ERR854913](https://www.ncbi.nlm.nih.gov/sra/ERR854913) | 12CFX_T_017 | 37053 | 250 |
| 181 | GC-17 | [ERR855357](https://www.ncbi.nlm.nih.gov/sra/ERR855357) | 12AZI_T_007 | 37512 | N |
| 182 | GC-18 | [ERR854870](https://www.ncbi.nlm.nih.gov/sra/ERR854870) | 12CFX_T_047 | 37083 | 90 |
| 183 | GC-19 | [ERR854902](https://www.ncbi.nlm.nih.gov/sra/ERR854902) | 12CFX_T_006 | 37042 | 90 |
| 184 | GC-20 | [ERR854916](https://www.ncbi.nlm.nih.gov/sra/ERR854916) | 12CFX_T_020 | 37056 | 90 |
| 185 | GC-21 | [ERR854898](https://www.ncbi.nlm.nih.gov/sra/ERR854898) | 12CFX_T_002 | 37038 | 90 |
| 186 | GC-22 | [ERR854919](https://www.ncbi.nlm.nih.gov/sra/ERR854919) | 12CFX_T_023 | 37059 | 90 |
| 187 | GC-23 | [ERR855360](https://www.ncbi.nlm.nih.gov/sra/ERR855360) | 12AZI_T_010 | 37515 | N |
| 188 | GC-24 | [ERR854932](https://www.ncbi.nlm.nih.gov/sra/ERR854932) | 12CFX_T_037 | 37073 | 250 |
| 189 | GC-25 | [ERR854903](https://www.ncbi.nlm.nih.gov/sra/ERR854903) | 12CFX_T_007 | 37043 | 90 |
| 190 | GC-26 | [ERR854936](https://www.ncbi.nlm.nih.gov/sra/ERR854936) | 12CFX_T_041 | 37077 | N |
| 191 | GC-27 | [ERR854899](https://www.ncbi.nlm.nih.gov/sra/ERR854899) | 12CFX_T_003 | 37039 | 250 |
| 192 | GC-28 | [ERR854917](https://www.ncbi.nlm.nih.gov/sra/ERR854917) | 12CFX_T_021 | 37057 | 90 |
| 193 | GC-29 | [ERR855351](https://www.ncbi.nlm.nih.gov/sra/ERR855351) | 12AZI_T_001 | 37506 | N |
| 194 | GC-30 | [ERR854905](https://www.ncbi.nlm.nih.gov/sra/ERR854905) | 12CFX_T_009 | 37045 | 88 |
| 195 | GC-31 | [ERR854928](https://www.ncbi.nlm.nih.gov/sra/ERR854928) | 12CFX_T_033 | 37069 | 90 |
| 196 | GC-32 | [ERR854927](https://www.ncbi.nlm.nih.gov/sra/ERR854927) | 12CFX_T_032 | 37068 | 246 |
| 197 | GC-33 | [ERR855353](https://www.ncbi.nlm.nih.gov/sra/ERR855353) | 12AZI_T_003 | 37508 | 250 |
| 198 | GC-34 | [ERR854904](https://www.ncbi.nlm.nih.gov/sra/ERR854904) | 12CFX_T_008 | 37044 | 90 |
| 199 | GC-35 | [ERR855358](https://www.ncbi.nlm.nih.gov/sra/ERR855358) | 12AZI_T_008 | 37513 | N |
| 200 | GC-36 | [ERR854929](https://www.ncbi.nlm.nih.gov/sra/ERR854929) | 12CFX_T_034 | 37070 | 90 |
| 201 | GC-37 | [ERR854912](https://www.ncbi.nlm.nih.gov/sra/ERR854912) | 12CFX_T_016 | 37052 | 90 |
| 202 | GC-38 | [ERR855359](https://www.ncbi.nlm.nih.gov/sra/ERR855359) | 12AZI_T_009 | 37514 | N |
| 203 | GC-39 | [ERR854908](https://www.ncbi.nlm.nih.gov/sra/ERR854908) | 12CFX_T_012 | 37048 | 90 |
| 204 | GC-40 | [ERR854867](https://www.ncbi.nlm.nih.gov/sra/ERR854867) | 12CFX_T_024 | 37060 | 90 |
| 205 | GC-41 | [ERR956689](https://www.ncbi.nlm.nih.gov/sra/ERR956689) | 12CFX_T_039 | 58867 | 90 |
| 206 | GC-42 | [ERR956690](https://www.ncbi.nlm.nih.gov/sra/ERR956690) | 12CFX_T_030 | 58866 | 250 |
| 207 | GC-43 | [ERR855324](https://www.ncbi.nlm.nih.gov/sra/ERR855324) | 12AZI_C_009 | 37479 | 90 |
| 208 | GC-44 | [ERR854930](https://www.ncbi.nlm.nih.gov/sra/ERR854930) | 12CFX_T_035 | 37071 | 90 |
| 209 | GC-45 | [ERR854933](https://www.ncbi.nlm.nih.gov/sra/ERR854933) | 12CFX_T_038 | 37074 | 90 |
| 210 | GC-46 | [ERR854911](https://www.ncbi.nlm.nih.gov/sra/ERR854911) | 12CFX_T_015 | 37051 | 90 |
| 211 | GC-47 | [ERR854910](https://www.ncbi.nlm.nih.gov/sra/ERR854910) | 12CFX_T_014 | 37050 | 90 |
| 212 | GC-48 | [ERR854939](https://www.ncbi.nlm.nih.gov/sra/ERR854939) | 12CFX_T_046 | 37082 | 90 |
| 213 | GC-49 | [ERR854920](https://www.ncbi.nlm.nih.gov/sra/ERR854920) | 12CFX_T_025 | 37061 | 250 |
| 214 | GC-50 | [ERR854935](https://www.ncbi.nlm.nih.gov/sra/ERR854935) | 12CFX_T_040 | 37076 | 90 |

N = not identified.

|  | **Allele** | **No. isolates** | **%** |
| --- | --- | --- | --- |
|  | 90 | 27 | 54 |
|  | 250 | 11 | 22 |
|  | N | 9 | 18 |
|  | 88 | 1 | 2 |
|  | 136 | 1 | 2 |
|  | 246 | 1 | 2 |
| Total |  | 50 | 100 |
