# Supplementary figures and images for: Antigenicity in mice of a recombinant Neisseria gonorrhoeae MafA 2/3 protein
Source: Virulence. 2025 Oct 29;16(1):2580086. doi: 10.1080/21505594.2025.2580086 (PMC12574560; doi:10.1080/21505594.2025.2580086)

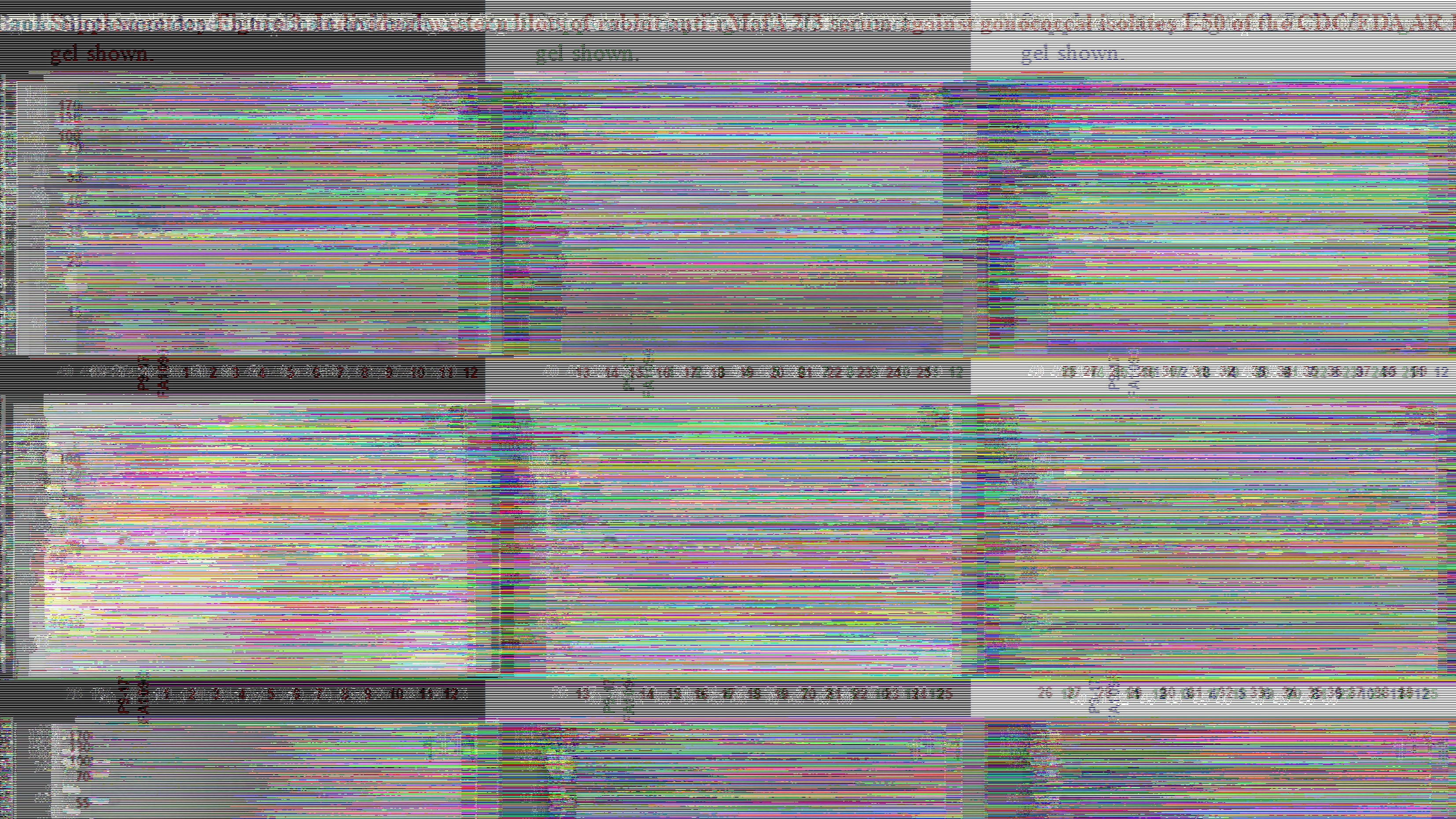

Supplement: Supplementary_Figure_3.tif [file KVIR_A_2580086_SM4703.tif]

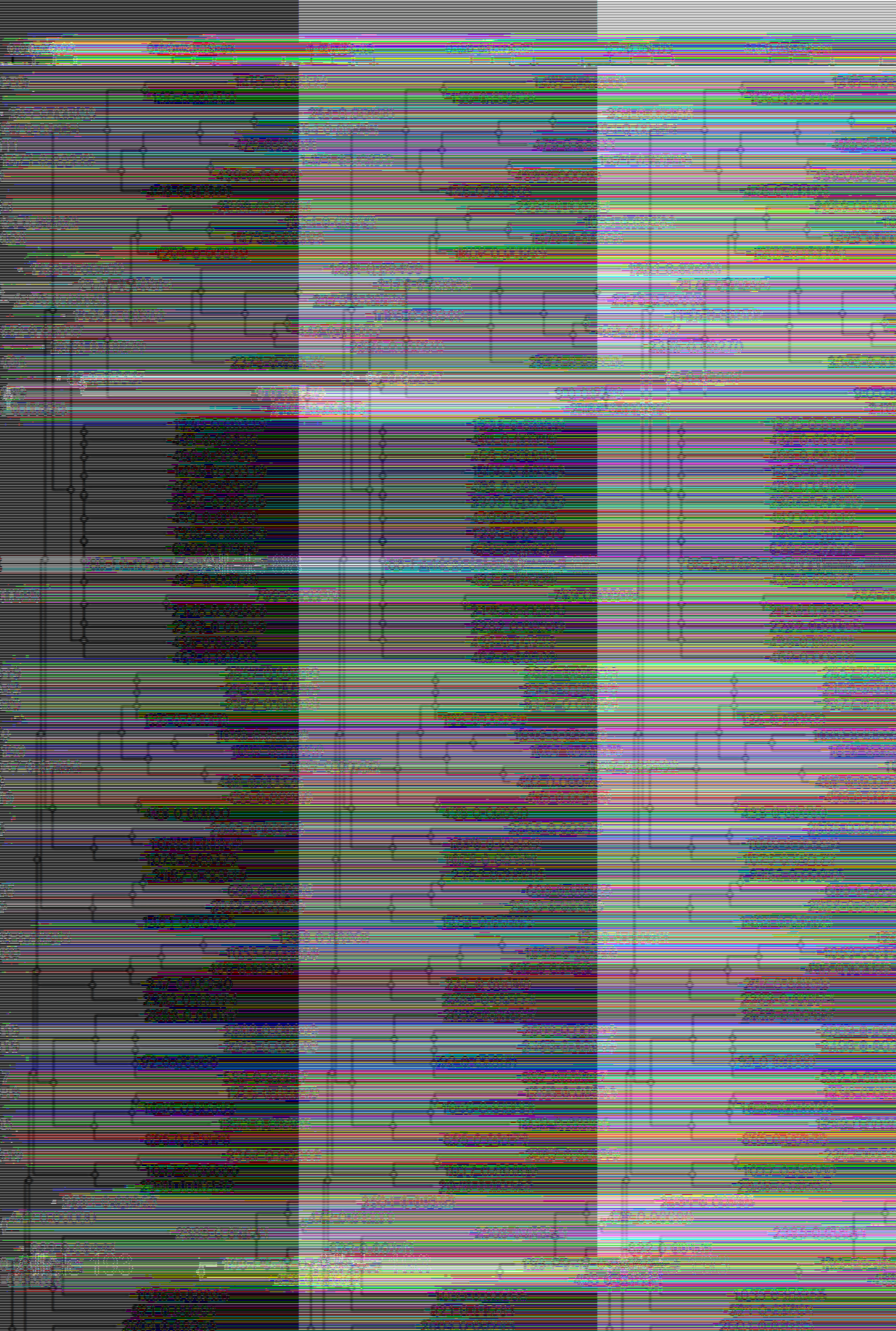

Supplement: Supplementary_Figure_2.tif [file KVIR_A_2580086_SM4699.tif]
