# Supplementary material for: Antigenicity in mice of a recombinant Neisseria gonorrhoeae MafA 2/3 protein
Source: Virulence. 2025 Oct 29;16(1):2580086. doi: 10.1080/21505594.2025.2580086 (PMC12574560; doi:10.1080/21505594.2025.2580086)
Supplement: Supplementary_Table2_revised.docx [file KVIR_A_2580086_SM4702.docx]

**Supplementary Table 2. Statistics for ELISA data.** Data were analysed using one-way ANOVA with Tukey’s multiple comparison test, or paired sample t-Test. P values <0.05 are considered significant.

**Figure 6. Antisera raised to rMafA 2/3 tested for reactivity against OM preparations**

1. **rMafA 2/3 sera against OM preparations**

|  | **Sera raised to rMafA 2/3 tested against** | | |
| --- | --- | --- | --- |
| **Sera raised to rMafA 2/3 in** | **P9-17 OM**  **(Allele 193)** | **FA1090 OM**  **(Allele 88)** | **AR205 OM**  **(Allele 90)** |
| Saline | 0.0038 | <0.0001 | <0.0001 |
| Alum | <0.0001 | <0.0001 | <0.0001 |
| Liposomes | 0.0078 | <0.0001 | <0.0001 |
| Liposomes+MPLA | <0.0001 | <0.0001 | <0.0001 |
| ZW 3-14 micelles | <0.0001 | <0.0001 | <0.0001 |
| Zw 3-14+MPLA | <0.0001 | <0.0001 | <0.0001 |

1. **Comparing reactivity of rMafA 2/3 between different OM preparations**

|  | **Sera raised to rMafA 2/3 tested against** | | |
| --- | --- | --- | --- |
| **Sera raised to rMafA 2/3 in** | **P9-17 OM (Allele 193) compared with FA1090 OM (Allele 88)** | **P9-17 OM (Allele 193) compared with AR205 OM (Allele 90)** | **FA1090 (Allele 88) compared with AR205 (Allele 90)** |
| Saline | 0.0001 | 0.0002 | >0.9999 |
| Alum | 0.0003 | 0.0002 | >0.9999 |
| Liposomes | <0.00001 | 0.0001 | >0.9999 |
| Liposomes+MPLA | <0.00001 | <0.00001 | 0.4389 |
| Zw 3-14 micelles | 0.0844 | 0.6363 | >0.9999 |
| Zw 3-14+MPLA | <0.00001 | <0.00001 | >0.9999 |

1. **Comparing the reactivities of serum groups against P9-17, FA1090 and AR205 bacteria in flow cytometry using a paired samples t-Test**

|  | **Paired Differences** | | | | | | | **Significance** | |
| --- | --- | --- | --- | --- | --- | --- | --- | --- | --- |
|  | **Mean** | **StDev** | **SEM** | **95% Confidence Interval of the Difference** | | **t** | **df** | **One-Sided p** | **Two-Sided p** |
|  |  |  |  | **Lower** | **Upper** |  |  |  |  |
| **P9-17-FA1090** | 1.36667 | 11.73093 | 4.78913 | -10.94419 | 13.67752 | 0.285 | 5 | 0.393 | 0.787 |
| **P9-17-AR205** | 61.8 | 10.86875 | 4.43715 | 50.39395 | 73.20605 | 13.928 | 5 | <.001 | <.001 |
| **FA1090-AR205** | 60.43333 | 14.93861 | 6.09866 | 44.75623 | 76.11044 | 9.909 | 5 | <.001 | <.001 |

1. **Statistics for IgG1 and IgG2a isotypes measured in ELISA, supporting Figure 11.**

**Comparing Th1 responses:**

| **Tukey's multiple comparisons test** | **Mean Diff.** | **95.00% CI of diff.** | **Below threshold?** | **Summary** | **Adjusted P Value** |
| --- | --- | --- | --- | --- | --- |
| Saline/IgG2a vs. Alum/IgG2a | 1.237 | -0.06059 to 2.535 | No | ns | 0.0670 |
| Saline/IgG2a vs. Liposomes/IgG2a | 0.5547 | -0.6689 to 1.778 | No | ns | 0.7080 |
| Saline/IgG2a vs. Liposomes+MPLA/IgG2a | -0.3287 | -1.552 to 0.8948 | No | ns | 0.9540 |
| Saline/IgG2a vs. Zwittergent3-14/IgG2a | 0.3312 | -1.082 to 1.744 | No | ns | 0.9741 |
| Saline/IgG2a vs. Zwittergent3-14+MPLA/IgG2a | -0.5289 | -1.942 to 0.8839 | No | ns | 0.8395 |
| Alum/IgG2a vs. Liposomes/IgG2a | -0.6825 | -1.980 to 0.6153 | No | ns | 0.5710 |
| Alum/IgG2a vs. Liposomes+MPLA/IgG2a | -1.566 | -2.864 to -0.2682 | Yes | * | 0.0128 |
| Alum/IgG2a vs. Zwittergent3-14/IgG2a | -0.9060 | -2.384 to 0.5716 | No | ns | 0.4112 |
| Alum/IgG2a vs. Zwittergent3-14+MPLA/IgG2a | -1.766 | -3.244 to -0.2885 | Yes | * | 0.0138 |
| Liposomes/IgG2a vs. Liposomes+MPLA/IgG2a | -0.8834 | -2.107 to 0.3401 | No | ns | 0.2491 |
| Liposomes/IgG2a vs. Zwittergent3-14/IgG2a | -0.2235 | -1.636 to 1.189 | No | ns | 0.9956 |
| Liposomes/IgG2a vs. Zwittergent3-14+MPLA/IgG2a | -1.084 | -2.496 to 0.3292 | No | ns | 0.1975 |
| Liposomes+MPLA/IgG2a vs. Zwittergent3-14/IgG2a | 0.6599 | -0.7529 to 2.073 | No | ns | 0.6827 |
| Liposomes+MPLA/IgG2a vs. Zwittergent3-14+MPLA/IgG2a | -0.2002 | -1.613 to 1.213 | No | ns | 0.9974 |
| Zwittergent3-14/IgG2a vs. Zwittergent3-14+MPLA/IgG2a | -0.8601 | -2.440 to 0.7195 | No | ns | 0.5357 |

**Comparing Th2 responses:**

| **Tukey's multiple comparisons test** | **Mean Diff.** | **95.00% CI of diff.** | **Below threshold?** | **Summary** | **Adjusted P Value** |
| --- | --- | --- | --- | --- | --- |
| Saline/IgG1 vs. Alum/ IgG1 | -0.4034 | -1.420 to 0.6131 | No | ns | 0.8087 |
| Saline/IgG1 vs. Liposomes/IgG1 | 0.6094 | -0.4071 to 1.626 | No | ns | 0.4392 |
| Saline/IgG1 vs. Liposomes+MPLA/IgG1 | -0.02776 | -1.044 to 0.9888 | No | ns | >0.9999 |
| Saline/IgG1 vs. Zwittergent3-14/IgG1 | 0.06349 | -1.110 to 1.237 | No | ns | >0.9999 |
| Saline/IgG1 vs. Zwittergent 3-14 +MPLA/IgG1 | -0.7244 | -1.898 to 0.4494 | No | ns | 0.4085 |
| Alum/ IgG1 vs. Liposomes/IgG1 | 1.013 | -0.003707 to 2.029 | No | ns | 0.0512 |
| Alum/ IgG1 vs. Liposomes+MPLA/IgG1 | 0.3757 | -0.6409 to 1.392 | No | ns | 0.8494 |
| Alum/ IgG1 vs. Zwittergent3-14/IgG1 | 0.4669 | -0.7069 to 1.641 | No | ns | 0.8072 |
| Alum/ IgG1 vs. Zwittergent 3-14 +MPLA/IgG1 | -0.3210 | -1.495 to 0.8528 | No | ns | 0.9518 |
| Liposomes/IgG1 vs. Liposomes+MPLA/IgG1 | -0.6372 | -1.654 to 0.3794 | No | ns | 0.3921 |
| Liposomes/IgG1 vs. Zwittergent3-14/IgG1 | -0.5459 | -1.720 to 0.6279 | No | ns | 0.6907 |
| Liposomes/IgG1 vs. Zwittergent 3-14 +MPLA/IgG1 | -1.334 | -2.508 to -0.1600 | Yes | * | 0.0203 |
| Liposomes+MPLA/IgG1 vs. Zwittergent3-14/IgG1 | 0.09125 | -1.083 to 1.265 | No | ns | 0.9999 |
| Liposomes+MPLA/IgG1 vs. Zwittergent 3-14 +MPLA/IgG1 | -0.6966 | -1.870 to 0.4772 | No | ns | 0.4499 |
| Zwittergent3-14/IgG1 vs. Zwittergent 3-14 +MPLA/IgG1 | -0.7879 | -2.100 to 0.5245 | No | ns | 0.4377 |

**Comparing Th1/Th2 of each adjuvant (Multiple comparison):**

| **Tukey's multiple comparisons test** | **Mean Diff.** | **95.00% CI of diff.** | **Below threshold?** | **Summary** | **Adjusted P Value** |
| --- | --- | --- | --- | --- | --- |
| Saline/IgG1 vs. Alum/ IgG1 | -0.4034 | -1.640 to 0.8327 | No | ns | 0.9910 |
| Saline/IgG1 vs. Liposomes/IgG1 | 0.6094 | -0.6267 to 1.846 | No | ns | 0.8519 |
| Saline/IgG1 vs. Liposomes+MPLA/IgG1 | -0.02776 | -1.264 to 1.208 | No | ns | >0.9999 |
| Saline/IgG1 vs. Zwittergent3-14/IgG1 | 0.06349 | -1.364 to 1.491 | No | ns | >0.9999 |
| Saline/IgG1 vs. Zwittergent 3-14 +MPLA/IgG1 | -0.7244 | -2.152 to 0.7030 | No | ns | 0.8275 |
|  |  |  |  |  |  |
| Saline/IgG1 vs. Saline/IgG2a | 1.562 | 0.3260 to 2.798 | Yes | ** | 0.0042 |
| Saline/IgG1 vs. Alum/IgG2a | 2.799 | 1.488 to 4.110 | Yes | **** | <0.0001 |
| Saline/IgG1 vs. Liposomes/IgG2a | 2.117 | 0.8807 to 3.353 | Yes | **** | <0.0001 |
| Saline/IgG1 vs. Liposomes+MPLA/IgG2a | 1.233 | -0.002720 to 2.470 | No | ns | 0.0509 |
| Saline/IgG1 vs. Zwittergent3-14/IgG2a | 1.893 | 0.4660 to 3.321 | Yes | ** | 0.0022 |
| Saline/IgG1 vs. Zwittergent3-14+MPLA/IgG2a | 1.033 | -0.3941 to 2.461 | No | ns | 0.3616 |
| Alum/ IgG1 vs. Liposomes/IgG1 | 1.013 | -0.2233 to 2.249 | No | ns | 0.2011 |
| Alum/ IgG1 vs. Liposomes+MPLA/IgG1 | 0.3757 | -0.8605 to 1.612 | No | ns | 0.9950 |
| Alum/ IgG1 vs. Zwittergent3-14/IgG1 | 0.4669 | -0.9604 to 1.894 | No | ns | 0.9908 |
| Alum/ IgG1 vs. Zwittergent 3-14 +MPLA/IgG1 | -0.3210 | -1.748 to 1.106 | No | ns | 0.9997 |
| Alum/ IgG1 vs. Saline/IgG2a | 1.966 | 0.7294 to 3.202 | Yes | *** | 0.0001 |
| Alum/ IgG1 vs. Alum/IgG2a | 3.203 | 1.892 to 4.514 | Yes | **** | <0.0001 |
| Alum/ IgG1 vs. Liposomes/IgG2a | 2.520 | 1.284 to 3.756 | Yes | **** | <0.0001 |
| Alum/ IgG1 vs. Liposomes+MPLA/IgG2a | 1.637 | 0.4007 to 2.873 | Yes | ** | 0.0023 |
| Alum/ IgG1 vs. Zwittergent3-14/IgG2a | 2.297 | 0.8694 to 3.724 | Yes | *** | 0.0001 |
| Alum/ IgG1 vs. Zwittergent3-14+MPLA/IgG2a | 1.437 | 0.009283 to 2.864 | Yes | * | 0.0473 |
| Liposomes/IgG1 vs. Liposomes+MPLA/IgG1 | -0.6372 | -1.873 to 0.5989 | No | ns | 0.8134 |
| Liposomes/IgG1 vs. Zwittergent3-14/IgG1 | -0.5459 | -1.973 to 0.8814 | No | ns | 0.9699 |
| Liposomes/IgG1 vs. Zwittergent 3-14 +MPLA/IgG1 | -1.334 | -2.761 to 0.09354 | No | ns | 0.0859 |
| Liposomes/IgG1 vs. Saline/IgG2a | 0.9527 | -0.2834 to 2.189 | No | ns | 0.2752 |
| Liposomes/IgG1 vs. Alum/IgG2a | 2.190 | 0.8788 to 3.501 | Yes | **** | <0.0001 |
| Liposomes/IgG1 vs. Liposomes/IgG2a | 1.507 | 0.2713 to 2.744 | Yes | ** | 0.0066 |
| Liposomes/IgG1 vs. Liposomes+MPLA/IgG2a | 0.6240 | -0.6121 to 1.860 | No | ns | 0.8323 |
| Liposomes/IgG1 vs. Zwittergent3-14/IgG2a | 1.284 | -0.1434 to 2.711 | No | ns | 0.1129 |
| Liposomes/IgG1 vs. Zwittergent3-14+MPLA/IgG2a | 0.4238 | -1.004 to 1.851 | No | ns | 0.9959 |
| Liposomes+MPLA/IgG1 vs. Zwittergent3-14/IgG1 | 0.09125 | -1.336 to 1.519 | No | ns | >0.9999 |
| Liposomes+MPLA/IgG1 vs. Zwittergent 3-14 +MPLA/IgG1 | -0.6966 | -2.124 to 0.7307 | No | ns | 0.8597 |
| Liposomes+MPLA/IgG1 vs. Saline/IgG2a | 1.590 | 0.3538 to 2.826 | Yes | ** | 0.0034 |
| Liposomes+MPLA/IgG1 vs. Alum/IgG2a | 2.827 | 1.516 to 4.138 | Yes | **** | <0.0001 |
| Liposomes+MPLA/IgG1 vs. Liposomes/IgG2a | 2.145 | 0.9085 to 3.381 | Yes | **** | <0.0001 |
| Liposomes+MPLA/IgG1 vs. Liposomes+MPLA/IgG2a | 1.261 | 0.02504 to 2.497 | Yes | * | 0.0420 |
| Liposomes+MPLA/IgG1 vs. Zwittergent3-14/IgG2a | 1.921 | 0.4937 to 3.348 | Yes | ** | 0.0018 |
| Liposomes+MPLA/IgG1 vs. Zwittergent3-14+MPLA/IgG2a | 1.061 | -0.3664 to 2.488 | No | ns | 0.3240 |
| Zwittergent3-14/IgG1 vs. Zwittergent 3-14 +MPLA/IgG1 | -0.7879 | -2.384 to 0.8079 | No | ns | 0.8507 |
| Zwittergent3-14/IgG1 vs. Saline/IgG2a | 1.499 | 0.07130 to 2.926 | Yes | * | 0.0323 |
| Zwittergent3-14/IgG1 vs. Alum/IgG2a | 2.736 | 1.243 to 4.229 | Yes | **** | <0.0001 |
| Zwittergent3-14/IgG1 vs. Liposomes/IgG2a | 2.053 | 0.6260 to 3.481 | Yes | *** | 0.0007 |
| Zwittergent3-14/IgG1 vs. Liposomes+MPLA/IgG2a | 1.170 | -0.2574 to 2.597 | No | ns | 0.2007 |
| Zwittergent3-14/IgG1 vs. Zwittergent3-14/IgG2a | 1.830 | 0.2340 to 3.426 | Yes | * | 0.0133 |
| Zwittergent3-14/IgG1 vs. Zwittergent3-14+MPLA/IgG2a | 0.9697 | -0.6261 to 2.566 | No | ns | 0.6189 |
| Zwittergent 3-14 +MPLA/IgG1 vs. Saline/IgG2a | 2.287 | 0.8592 to 3.714 | Yes | *** | 0.0001 |
| Zwittergent 3-14 +MPLA/IgG1 vs. Alum/IgG2a | 3.524 | 2.031 to 5.016 | Yes | **** | <0.0001 |
| Zwittergent 3-14 +MPLA/IgG1 vs. Liposomes/IgG2a | 2.841 | 1.414 to 4.269 | Yes | **** | <0.0001 |
| Zwittergent 3-14 +MPLA/IgG1 vs. Liposomes+MPLA/IgG2a | 1.958 | 0.5304 to 3.385 | Yes | ** | 0.0014 |
| Zwittergent 3-14 +MPLA/IgG1 vs. Zwittergent3-14/IgG2a | 2.618 | 1.022 to 4.214 | Yes | **** | <0.0001 |
| Zwittergent 3-14 +MPLA/IgG1 vs. Zwittergent3-14+MPLA/IgG2a | 1.758 | 0.1618 to 3.353 | Yes | * | 0.0203 |
| Saline/IgG2a vs. Alum/IgG2a | 1.237 | -0.07390 to 2.548 | No | ns | 0.0798 |
| Saline/IgG2a vs. Liposomes/IgG2a | 0.5547 | -0.6814 to 1.791 | No | ns | 0.9135 |
| Saline/IgG2a vs. Liposomes+MPLA/IgG2a | -0.3287 | -1.565 to 0.9074 | No | ns | 0.9984 |
| Saline/IgG2a vs. Zwittergent3-14/IgG2a | 0.3312 | -1.096 to 1.759 | No | ns | 0.9995 |
| Saline/IgG2a vs. Zwittergent3-14+MPLA/IgG2a | -0.5289 | -1.956 to 0.8984 | No | ns | 0.9761 |
| Alum/IgG2a vs. Liposomes/IgG2a | -0.6825 | -1.994 to 0.6286 | No | ns | 0.8040 |
| Alum/IgG2a vs. Liposomes+MPLA/IgG2a | -1.566 | -2.877 to -0.2548 | Yes | ** | 0.0084 |
| Alum/IgG2a vs. Zwittergent3-14/IgG2a | -0.9060 | -2.399 to 0.5867 | No | ns | 0.6205 |
| Alum/IgG2a vs. Zwittergent3-14+MPLA/IgG2a | -1.766 | -3.259 to -0.2734 | Yes | ** | 0.0093 |
| Liposomes/IgG2a vs. Liposomes+MPLA/IgG2a | -0.8834 | -2.120 to 0.3527 | No | ns | 0.3801 |
| Liposomes/IgG2a vs. Zwittergent3-14/IgG2a | -0.2235 | -1.651 to 1.204 | No | ns | >0.9999 |
| Liposomes/IgG2a vs. Zwittergent3-14+MPLA/IgG2a | -1.084 | -2.511 to 0.3437 | No | ns | 0.2951 |
| Liposomes+MPLA/IgG2a vs. Zwittergent3-14/IgG2a | 0.6599 | -0.7674 to 2.087 | No | ns | 0.8966 |
| Liposomes+MPLA/IgG2a vs. Zwittergent3-14+MPLA/IgG2a | -0.2002 | -1.628 to 1.227 | No | ns | >0.9999 |
| Zwittergent3-14/IgG2a vs. Zwittergent3-14+MPLA/IgG2a | -0.8601 | -2.456 to 0.7357 | No | ns | 0.7685 |
