# Supplementary material for: Antigenicity in mice of a recombinant Neisseria gonorrhoeae MafA 2/3 protein
Source: Virulence. 2025 Oct 29;16(1):2580086. doi: 10.1080/21505594.2025.2580086 (PMC12574560; doi:10.1080/21505594.2025.2580086)
Supplement: Supplementary_Figure_1.docx [file KVIR_A_2580086_SM4701.docx]

**Supplementary Figure 1. Clustal alignment of the 101 non-redundant MafA 2/3 (NEIS0596) allele amino acid sequences**

1467 MKTLLLLIPLVLTACGTLTGIPAHGGGKRFAVEQELVVASSRAAVKEMDLSALKGRKAAL 60

251 MKILLLLIPLVLTACGTLTGIPAHGGGKRFAVEQELVAASSRAAVKEMDLSALKGRKAAL 60

475 MKTLLLLIPLVLTACGTLTGIPAHGGGKRFAVEQELVAASSRAAVKEMDLSALKGRKAAL 60

196 MKTLLLLIPLVLTACGTLTGIPAHGGGKRFAVEQELVAASSRAAVKEMDLSALKGRKAAL 60

1471 MKILLLLIPLVLTACGTLTGIPAHGGGKRFAVEQELVAASSRAAVKEMDLSALKGRKAAL 60

464 MKILLLLIPLVLTACGTLTGIPAHGGGKRFAVEQELVAASSRAAVKEMDLSALKGRKAAL 60

439 MKILLLLIPLVLTACGTLTGIPAHGGGKRFAVEQELVAASSRAAVKEMDLSALKGRKAAL 60

446 MKTLLLLIPLVLTACGTLTGIPAHGGGKRFAVEQELVAASSRAAVKEMDLSALKGRKAAL 60

2501 -MKILLLIPLVLTACGTLTGIPAHGGGKRFAVEQELVAASSRAAVKKMDLSALKGRKAAL 59

822 -MKILLLIPLVLTACGTLTGIPAHGGGKRFAVEQELVAASSRAAVKEMDLSALKGRKAAL 59

472 -MKILLLIPLVLTACGTLTGIPAHGGGKRFAVEQELVAASSRAAVKEMDLSALKGRKAAL 59

2657 MKTLLLLIPLVLTACGTLTGIPAHGGGKRFAVEQELVAASSRAAVKKMDLSALKGRKAAL 60

2369 MKTLLLLIPLVLTACGTLTGIPAHGGGKRFAVEQELVAASSRAAVKKMDLSALKGRKAAL 60

2143 MKTLLLLIPLVLTACGTLTGIPAHGGGKRFAVEQELVAASSRAAVKKMDLSALKGRKAAL 60

2665 MKTLLLLIPLVLTACGTLTGIPAHGGGKRFAVEQELVAASSRAAVKKMDLSALKGRKAAL 60

1048 MKTLLLLIPLVLTACGTLTGIPAHGGGKRFAVEQELVAASSRAAVKKMDLSALKGRKAAL 60

2477 MKTLLLLIPLVLTACGTLTGIPAHGGGKRFAVEQELVAASSRAAVKKMDLSALKGRKAAL 60

1047 MKTLLLLIPLVLTACGTLTGIPAHGGGKRFAVEQELVAASSRAAVKKMDLSALKGRKAAL 60

1238 MKTLLLLIPLVLTACGTLTGIPAHGGGKRFAVEQELVAASSRAAVKKMDLSALKGRKAAL 60

452 MKTLLLLIPLVLTACGTLTGIPAHGGGKRFAVEQELVAASSRAAVKKMDLSALKGRKAAL 60

456 MKTLLLLIPLVLTACGTLTGIPAHGGGKRFAVEQELVAASSRAAVKKMDLSALKGRKAAL 60

136 MKTLLLLIPLVLTACGTLTGIPAHGGGKRFAVEQELVAASSRAAVKKMDLSALKGRKAAL 60

2077 MKTLLLLIPLVLTACGTLTGIPAHGGGKRFAVEQELVAASSRAAVKEMDLSALKGRKAAL 60

1201 MKTLLLLIPLVLTACGTLTGIPAHGGGKRFAVEQELVAASSRAAVKEMDLSALKGRKAAL 60

2176 MKTLLLLIPLVLTACGTLTGIPAHGGGKRFAVEQELVAASSRAAVKEMDLSALKGRKAAL 60

2175 MKTLLLLIPLVLTACGTLTGIPAHGGGKRFAVEQELVAASSRAAVKEMDLSALKGRKAAL 60

2702 MKTLLLLIPLVLTACGTLTGVPAHGGGKRFAVEQELVAASSRAAVKEMDLSALKGRKAAL 60

2653 MKTLLLLIPLVLTACGTLTGIPAHGGGKRFAVEQELVAASSRAAVKEMDLSALKGRKAAL 60

2604 MKTLLLLIPLVLTACGTLTGIPAHGGGKRFAVEQELVAASSRAAVKEMDLSALKGRKAAL 60

2603 MKTLLLLIPLVLTACGTLTGIPAHGGGKRFAVEQELVAASSRAAVKEMDLSALKGRKAAL 60

2599 MKTLLLLIPLVLTACGTLTGIPAHGGGKRFAVEQELVAASSRAAVKEMDLSALKGRKAAL 60

2496 MKTLLLFIPLVLTACGTLTGIPAHGGGKRFAVEQELVAASSRAAVKEMDLSALKGRKAAL 60

2375 MKTLLLLIPLVLTACGTLTGIPAHGGGKRFAVEQELVAASSRAAVKEMDLSALKGRKAAL 60

2347 MKTLLLLIPLVLTACGTLTGIPAHGGGKRFAVEQELVAASSRAAVKEMDLSALKGCKAAL 60

2170 MKTLLLLIPLVLTACGTLTGIPAHGGGKRFAVEQELVAASSRAAVKEMDLSALKGRKAAL 60

2152 MKTLLLLIPLVLTACGTLTGIPAHGGGKRFAVEQELVAASSRAAVKEMDLSALKGRKAAL 60

2083 MKILLLLIPLVLTACGTLTGIPAHGGGKRFAVEQELVAASSRAAVKEMDLSALKGRKAAL 60

1498 MKTLLLLIPLVLTACGTLTGIPAHGGGKRFAVEQELVAASSRAAVKEMDLSALKGRKAAL 60

1481 MKTLLLLIPLVLTACGTLTGIPAHGGGKRFAVEQELVAASSRAAVKEMDLSALKGRKAAL 60

1468 MKTLLLLIPLVLTACGTLTGIPAHGGGKRFAVEQELVAASSRAAVKEMDLSALKGRKAAL 60

1459 MKTLLLLIPLVLTACGTLTGIPAHGGGKRFAVEQELVAASSRAAVKEMDLSALKGRKAAL 60

1412 MKTLLLLIPLVLTACGTLTGIPAHGGGKRFAVEQELVAASSRAAVKEMDLSALKGRKAAL 60

1296 MKTLLLLIPLVLTACGTLTGIPAHGGGKRFAVEQELVAASSRAAVKEMDLSALKGRKAAL 60

1295 MKTLLLLTPLVLTACGTLTGIPAHGGGKRFAVEQELVAASSRAAVKEMDLSALKGRKAAL 60

1202 MKTLLLLIPLVLTACGTLTGIPAHGGGKRFAVEQELVAASSRAAVKEMDLSALKGRKAAL 60

1125 MKTLLLLIPLVLTACGTLTGIPAHGGGKRFAVEQELVAASSRAAVKEMDLSALKGRKAAL 60

1057 MKTLLLLIPLVLTACGTLTGIPAHGGGKRFAVEQELVAASSRAAVKKMDLSALKGRKAAL 60

678 MKTLLLLIPLVLTACGTLTGIPAHGGGKRFAVEQELVAASSRAAVKEMDLSALKGRKAAL 60

582 MKTLLLLIPLVLTACGTLTGIPAHGGGKRFAVEQELVAASSRAAVKEMDLSALKGRKAAL 60

469 MKTLLLLIPLVLTACGTLTGIPAHGGGKRFAVEQELVAASSRAAVKEMDLSALKGRKAAL 60

466 MKTLLLLIPLVLTACGTLTDIPAHGGGKRFAVEQELVAASSRAAVKEMDLSALKGRKAAL 60

462 MKTLLLLIPLVLTACGTLTGIPAHGGGKRFAVEQELVAASSRAAVKEMDLSALKGRKAAL 60

459 MKTLLLLIPLVLTACGTLTGIPAHGGGKRFAVEQELVAASSRAAIKEMDLSALKGRKAAL 60

437 MKTLLLLIPLVLTACGTLTGIPAHGGGKRFAVEQELVAASSRAAVKEMDLSALKGRKAAL 60

431 MKTLLLLIPLVLTACGTLTGIPAHGGGKRFAVEQELVAASSRAAVKEMDLSALKGRKATL 60

2367 MKTLLLLIPLVLTACGTLTGIPAHGGGKRFAVEQELVAASSRAAVKEMDLSALKGRKAAL 60

2326 MKTLLLLIPLVLTACGTLTGIPAHGGGKRFAVEQELVAASSRAAVKKMDLSALKGRKAAL 60

2283 MKTLLLLIPLVLTACGTLTGIPAHGGGKRFAVEQELVAASSRAAVKEMDLSALKGRKAAL 60

2282 MKTLLLLIPLVLTACGTLTGIPAHGGGKRFAVEQELVAASSRAAVKEMDLSALKGRKAAL 60

1456 MKTLLLLIPLVLTACGTLTGIPAHGGGKRFAVEQELVAASSRAAVKEMDLSALKGRKAAL 60

1242 MKTLLLLIPLVLTACGTLTGIPAHGGGKRFAVEQELVAASSRAAVKEMDLSALKGRKAAL 60

1055 MKPLLLLIPLVLTACGTLTGIPAHGGGKRFAVEQELVAASSRAAVKEMDLSALKGRKAAL 60

1054 MKTLLLLIPLVLTACGTLTGIPAHGGGKRFAVEQELVAASSRAAVKKMDLSALKGRKAAL 60

789 MKTLLLLIPLVLTACGTLTGIPAHGGGKRFAVEQELVAASSRAAVKEMDLSALKGRKAAL 60

600 MKTLLLLIPLVLTACGTLTGIPAHGGGKRFAVEQELVAASSRAAVKEMDLSALKGRKAAL 60

455 MKTLLLLIPLVLTACGTLTGIPAHGGGKRFAVEQELVAASSRAAVKKMDLSALKGRKAAL 60

451 MKTLLLLIPLVLTACGTLTGIPAHGGGKRFAVEQELVAASSRAAVKEMDLSALKGRKAAL 60

2337 MKTLLLLIPLVLTACGTLTGIPAHGGGKRFAVEQELVAASSRAAVKEMDLSALKGRKAAL 60

2089 MKTLLLLIPLVLTACGTLTGIPAHGGGKRFAVEQELVAASSRAAVKKMDLSALKGRKAAL 60

468 MKTLLLLIPLVLTACGTLTGIPAHGGGKRFAVEQELVAASSRAAVKEMDLSALKGRKAAL 60

442 MKTLLLLIPLVLTACGTLTGIPAHGGGKRFAVEQELVAASSRAAVKEMDLSALKGRKAAL 60

2040 MKTLLLLIPLVLTACGTLTGIPAHGGGKRFAVEQELVAASSRAAVKEMDLSALKGRKAAL 60

1075 MKTLLLLIPLVLTACGTLTGIPAHGGGKRFAVEQELVAASSRAAVKEMDLSALKGRKAAL 60

521 MKILLLLIPLVLTACGTLTGIPAHGGGKRFAVEQELVAASSRAAVKEMDLSALKGRKAAL 60

2305 MKTLLLLIPLVLTACGTLTGIPAHGGGKRFAVEQELVAASSRAAVKEMDLSALKGRKAAL 60

1473 MKTLLLLIPLVLTACGTLTGIPAHGGGKRFAVEQELVAASSRAAVKEMDLSALKGRKAAL 60

1284 MKTLLLLISLVLTACGTLTGIPAHGGGKRFAVEQELVAASSRAAVKEMDLSALKGRKAAL 60

679 MKTLLLLIPLVLTACGTLTGIPAHGGGKRFAVEQELFAASSRAAVKEMDLSALKGRKAAL 60

2146 MKTLLLLIPLVLTACGTLTGIPAHGGGKRFAVEQELVAASSRAAVKEMDLSALKGRKAAL 60

1046 MKTLLLLIPLVLTACGTLTGIPAHGGGKRFAVEQELVAASSRAAVKEMDLSALKGRKAAL 60

605 MKTLLLLIPLVLTACGTLTGIPAHGGGKRFAVEQELVAASSRAAVKEMDLSALKGRKAAL 60

1053 MKTLLLLIPLVLTACGTLTGIPAHGGGKRFAVEQELVAASSRAAVKEMDLSALKGRKAAL 60

855 MKTLLLLIPLVLTACGTLTGIPAHGGGKRFAVEQELVAASSRAAVKEMDLSALKGRKAAL 60

1388 MKTLLLLIPLVLTACGTLTGIPAHGGGKRFAVEQELVAASSRAAVKEMDLSALKGRKAAL 60

362 MKTLLLLIPLVLTACGTLTGIPAHGGGKRFAVEQELVAASSRAAVKEMDLSALKGRKAAL 60

438 MKTLLLLIPLVLTACGTLTGIPAHGGGKRFAVEQELVAASSRAAVKKMDLSALKGRKAAL 60

1017 MKTLLLLIPLVLTACGTLTGIPAHGGGKRFAVEQELVAASSRAAVKEMDLSALKGRKAAL 60

436 MKTLLLLIPLVLTACGTLTGIPAHGGGKRFAVEQELVAASSRAAVKEMDLSALKGRKAAL 60

1070 MKTLLLLIPLVLTACGTLTGIPAHGGGKRFAVEQELVAASSRAAVKEMDLSALKGRKAAL 60

92 MKTLLLLIPLVLTACGTLTGIPAHGGGKRFAVEQELVAASSRAAVKEMDLSALKGRKAAL 60

449 MKTLLLLIPLVLTACGTLTGIPAHGGGKRFAVEQELVAASSRAAVKEMDLSALKGRKAAL 60

247 MKTLLLLIPLVLTACGTLTGIPAHGGGKRFAVEQELVAASSRAAVKEMDLSALKGRKAAL 60

88-FA1090 MKTLLLLIPLVLTACGTLTGIPAHGGGKRFAVEQELVAASSRAAVKEMDLSALKGRKAAL 60

90-AR205 MKTLLLLIPLVLTACGTLTGIPAHGGGKRFAVEQELVAASSRAAVKEMDLSALKGRKAAL 60

460 MKTLLLLIPLVLTACGTLTGIPAHGGGKRFAVEQELVAASSRAAVKEMDLSALKGRKAAL 60

2385 -MKILLLIPLVLTACGTLTGIPAHGGGKRFAVEQELVAASSRAAVKEMDLSALKGRKAAL 59

193-P9-17 -MKILLLIPLVLTACGTLTGIPAHGGGKRFAVEQELVAASSRAAVKEMDLSALKGRKAAL 59

458 -MKILLLIPLVLTACGTLTGIPAHGGGKRFAVEQELVAASSRAAVKKMDLSALKGRKAAL 59

65 MKTLLLLIPLVLTACGTLTGIPAHGGGKRFAVEQELVAASSRAAVKEMDLSALKGHKAAL 60

9 MKILLLLIPLVLTACGTLTGIPAHGGGKRFAVEQELVAASSRAAVKEMDLSALKGRKAAL 60

2102 MKTLLLLIPLVLTACGTLTGIPAHGGGKRFAVEQELVAASSRAAVKEMDLSALKGRKAAL 60

:**: **********.:***************..******:*:******** **:*

1467 YVSVMGDQGSGNISGGRYSIDALIRGGYQNNPDSATRYSYPAYDTTATTKSDALSGVTTS 120

251 YVSVMGDQGSGNISGGRYSIDALIRGGYQNNPDSATRYSYPAYDTTATTKSDALSGVTTS 120

475 YVSVMGDQGSGNISGGRYSIDALIRGGYQNNPDSATRYSYPAYDTTATTKSDALSGVTTS 120

196 YVSVMGDQGSGNISGGRYSIDALIRGGYQNNPDSATRYSYPAYDTTATTKSDALSGVTTS 120

1471 YVSVMGDQGSGNISGGRYSIDALIRGGYQNNPDSATRYSYPAYDTTATTKSDALSGVTTS 120

464 YVSVMGDQGSGNISGGRYSIDALIRGGYQNNPDSATRYSYPAYDTTATTKSDALSGVTTS 120

439 YVSVMGDQGSGNISGGRYSIDALIRGGYQNNPDSATRYSYPAYDTTATTKSDALSGVTTS 120

446 YVSVMGDQGSGNISGGRYSIDALIRGGYQNNPDSATRYSYPAYDTTATTKSDALSGVTTS 120

2501 YVSVMGDQGSGNISGGRYSIDALIRGGYHNNPDSATRYSYPAYDTTATTKSDALSGVTTS 119

822 YVSVMGDQGSGNISGGRYSIDALIRGGYHNNPDSATRYSYPAYDTTATTKSDALSGVTTS 119

472 YVSVMGDQGSGNISGGRYSIDALIRGGYHNNPDSATRYSYPAYDTTATTKSDALSGVTTS 119

2657 YVSVMGDQGSGNISGGRYSIDALIRGGYHNNPDSATRYSYPAYDTTATTKSDALSGVTTS 120

2369 YVSVMGDQGSGNISGGRYSIDALIRGGYHNNPDSATRYSYPAYDTTATTKSDALSGVTTS 120

2143 YVSVMGDQGSCNISGGRYSIDALIRGGYHNNPDSATRYSYPAYDTTATTKSDALSGVTTS 120

2665 YVSVMGDQGSGNISGGRYSIDALIRGGYHNNPDSATRYSYPAYDTTATTKSDALSGVTTS 120

1048 YVSVMGDQGSGNISGGRYSIDALIRGGYHNNPDSATRYSSPAYDTTATTKSDALSGVTTS 120

2477 YVSVMGDQGSGNISGGRYSIDALIRGGYHNNPDSATRYSYPAYDTTATTKSDALSGVTTS 120

1047 YVSVMGDQGSGNISGGRYSIDALIRGGYQNNPDSATRYSSPAYDTTATTKSDALSGVTTS 120

1238 YVSVMGDQGSGNISGGRYSIDALIRGGYHNNPDSATRYSSPAYDTTATTKSDALSGVTTS 120

452 YVSVMGDQGSGNISGGRYSIDALIRGGYQNNPDSATRYSYPAYDTTATTKSDALSGVTTS 120

456 YVSVMGDQGSGNISGGRYSIDALIRGGYHNNPDSATRYSYPAYDTTATTKSDALSGVTTS 120

136 YVSVMGDQGSGNISGGRYSIDALIRGGYHNNPDSATRYSYPAYDTTATTKSDALSGVTTS 120

2077 YVSVMGDQGSGNISGGRYSIDALIRGGSHNTPDSATRYSYPAYDTTATTKSDALSGVTTS 120

1201 YVSVMGDQGSGNISGGRYSIDALIRGGYHNNPDSATRYSYPAYDTTATTKSDALSGVTTS 120

2176 YVSVMGDQGSGNISGGRYSIDALIRGGYQNNPDSATRYSYPAYDTTATTKSDALSGVTTS 120

2175 YVSVMGDQGSGNISGGRYSIDALIRGGYHNNPDSATRYSYPAYDTTATTKSDALSGVTTS 120

2702 YVSVMGDQGSGNISGGRYSIDALIRGGYHNNPDSATRYSYPAYDTTATTKSDALSGVTTS 120

2653 YVSVMGDQGSGNISGGRYSIDALIRGGYHNNPDSATRYSYPAYDTTATTKSDALSGVTTS 120

2604 YVSVMGDQGSGNISGGRYSIDALIRGGYHNNPDSATRYSYPAYDTTATTKSDALSGVTTS 120

2603 YVSVMGDQGSGNISGGRYSIDALIRGGYHNNPDSATRYSYPAYDTTATTKSDALSGVTTS 120

2599 YVSVMGDQGSGNISGGRYSIDALIRGGYHNNPDSATRYSYPAYDTTATTKSDALSGVTTS 120

2496 YVSVMGDQGSGNISGGRYSIDALIRGGYHNNPDSATRYSYPAYDTTATTKSDALSGVTTS 120

2375 YVSVMGDQGSGNISGGRYSIDALIRGGYHNNPDSATRYSYPAYDTTATTKSDALSGVTTS 120

2347 YVSVMGDQGSGNISGGRYSIDALIRGGYHNNPDSATRYSYPAYDTTATTKSDALSGVTTS 120

2170 YVSVMGDQGSGNISGGRYSIDALIRGGYHNNPDSATRYSYPAYDTTATTKSDALSGVTTS 120

2152 YVSVMGDQGSGNISGGRYSIDALIRGGYHNNPDSTTRYSYPAYDTTATTKSDALSGVTTS 120

2083 YVSVMGDQGSGNISGGRYSIDALIRGGYHNNPDSATRYSYPAYDTTATTKSDALSGVTTS 120

1498 YVSVMGDQGSGNISGGRYSIDALIRGGYHNNPDSATRYSYPAYDTTATTKSDALSGVTTS 120

1481 YVSVMGDQGSGNISGGRYSIDALIRGGYHNNPDSTTRYSYPAYDTTATTKSDALSGVTTS 120

1468 YVSVMGDQGSGNISGGRYSIDALIRGGYHNNPDSATRYSYPAYDTTATTKSDALSGVTTS 120

1459 YVSVMGDQGSGNISGGRYSIDALIRGGYHNNPDSATRYSYPAYDTTATTKSDALSGVTTS 120

1412 YVSVMGDQGSGNISGGRYSIDALIRGGYHNNPDSATRYSYPAYDTTATTKSDALSGVTTS 120

1296 YVSVMGDQGSGNISGGRYSIDALIRGGYHNNPDSAPRYSSPAYDTTATTKSDALSGVTTS 120

1295 YVSVMGDQGSGNISGGRYSIDALIRGGYHNNPDSATRYSYPAYDTTATTKSDALSGVTTS 120

1202 YVSVMGDQGSGNISGGRYSIDALIRGGYHNNPDSATRYSYPAYDTTATTKSDALSGVTTS 120

1125 YVSVMGDQGSGNISGGRYSIDALIRGGYHNNPDSATRYSYPAYDTTATTKSDALSGVTTS 120

1057 YVSVMGDQGSGNISGGRYSIDALIRGGYHNNPDSATRYSSPAYDTTATTKSDALSGVTTS 120

678 YVSVMGDQGSGNISGGRYSIDALIRGGYHNNPDSATRYSYPAYDTTATTKSDALSGVTTS 120

582 YVSVMGDQGSGNISGGRYSIDALIRGGYHNNPDSATRYSYPAYDTTATTKSDALSGVTTS 120

469 YVSVMGDQGSGNISGGRYSIDALIRGGYHNNPDSATRYSYPAYDTTATTKSDALSGVTTS 120

466 YVSVMGDQGSGNISGGRYSIDALIRGGYHNNPDSATRYSYPAYDTTATTKSDALSGVTTS 120

462 YVSVMGDQGSGNISGGRYSIDALIRGGYHNNPDSATRYSYPAYDTTATTKADALSGVTTS 120

459 YVSVMGDQGSGNISGGRYSIDALIRGGYHNNPDSATRYSYPAYDTTATTKSDALSGVTTS 120

437 YVSVMGDQGSGNISGGRYSIDALIRGGYHNNPDSATRYSYPAYDTTATTKSDALSGVTTS 120

431 YVSVMGDQGSGNISGGRYSIDALIRGGYHNNPDSATRYSYPAYDTTATTKSDALSGVTTS 120

2367 YVSVMGDQGSGNISGGRYSIDALIRGGYHNNPDSATRYSYPAYDTTATTKSDALSGVTTS 120

2326 YVSVMGDQGSGNISGGRYSIDALIRGGYHNNPDSATRYSYPAYDTTATTKSDALSGVTTS 120

2283 YVSVMGDQGSGNISGGRYSIDALIRGGYHNNPDSATRYSYPAYDTTATTKSDALSGVTTS 120

2282 YVSVMGDQGSGNISGGRYSIDALIRGGYHNNPDSATRYSYPAYDTTATTKSDALSGVTTS 120

1456 YVSVMGDQGSGNISGGRYSIDALIRGGYHNNPDSATRYSYPAYDTTATTKSDALSGVTTS 120

1242 YVSVMGDQGSGNISGGRYSIDALIRGGYHNNPDSATRYSSPAYDTTATTKSDALSGVTTS 120

1055 YVSVMGDQGSGNISGGRYSIDALIRGGYHNNPDSATRYSYPAYDTTATTKSDALSGVTTS 120

1054 YVSVMGDQGSGNISGGRYSIDALIRGGYHNNPDSATRYSYPAYDTTATTKSDALSGVTTS 120

789 YVSVMGDQGSGNISGGRYSIDALIRGGYHNNPDSATRYSYPAYDTTATTKSDALSGVTTS 120

600 YVSVMGDQGSGNISGGRYSIDALIRGGYHNNPDSTTRYSYPAYDTTATTKSDALSGVTTS 120

455 YVSVMGDQGSGNISGGRYSIDALIRGGYHNNPDSATRYSYPAYDTTATTKSDALSGVTTS 120

451 YVSVMGDQGSGNISGGRYSIDALIRGGYHNNPDSATRYSYPAYDTTATTKSDALSGVTTS 120

2337 YVSVMGDQGSGNISGGRYSIDALIRGGYHNNPDSATRYSYPAYDTTATTKSDALSGVTTS 120

2089 YVSVMGDQGSGNISGGRYSIDALIRGGYHNNPDSTTRYSYPAYDTTATTKSDALSGVTTS 120

468 YVSVMGDQGSGNISGGRYSIDALIRGGYHNNPDSATRYSYPAYDTTATTKSDALSGVTTS 120

442 YVSVMGDQGSGNISGGRYSIDALIRGGYHNNPDSATRYSYPAYDTTATTKSDALSGVTTS 120

2040 YVSVMGDQGSGNISGGRYSIDALIRGGYHNNPDSATRYSYPAYDTTATTKSDALSGVTTS 120

1075 YVSVMGDQGSGNISGGRYSIDALIRGGYHNNPDSATRYSYPAYDTTATTKSDALSGVTTS 120

521 YVSVMGDQGSGNISGGRYSIDALIRGGYHNNPDSATRYSYPAYDTTATTKSDALSGVTTS 120

2305 YVSVMGDQGSGNISGGRYSIDALIRGGYHNNPDSATRYSYPAYDTTATTKSDALSGVTTS 120

1473 YVSVMGDQGSGNISGGRYSIDALIRGGYHNNPDSATRYSYPAYDTTATTKSDALSGVTTS 120

1284 YVSVMGDQGSGNISGGRYSIDALIRGGYHNNPDSATRYSYPAYDTTATTKSDALSGVTTS 120

679 YVSVMGDQGSGNISGGRYSIDALIRGGYHNNPDSATRYSYPAYDTTATTKSDALSGVTTS 120

2146 YVSVMGDQGSGNISGGRYSIDALIRGGYHNNPDSATRYSYPAYDTTATTKSDALSGVTTS 120

1046 YVSVMGDQGSGNISGGRYSIDALIRGGYHNNPDSATRYSSPAYDTTATTKSDALSGVTTS 120

605 YVSVMGDQGSGNISGGRYSIDALIRGGYHNNPDSATRYSYPAYDTTATTKSDALSGVTTS 120

1053 YVSVMGDQGSGNISGGRYSIDALIRGGYHNNPDSATRYSSPAYDTTATTKSDALSGVTTS 120

855 YVSVMGDQGSGNISGGRYSIDALIRGGYHNNPDSATRYSSPAYDTTATTKSDALSGVTTS 120

1388 YVSVMGDQGSGNISGGRYSIDALIRGGYHNNPDSATRYSYPAYDTTATTKSDALSGVTTS 120

362 YVSVMGDQGSGNISGGRYSIDALIRGGYHNNPDSATRYSYPAYDTTATTKSDALSGVTTS 120

438 YVSVMGDQGSGNISGGRYSIDALIRGGYHNNPDSATRYSYPAYDTTATTKSDALSGVTTS 120

1017 YVSVMGDQGSGNISGGRYSIDALIRGGYHNNPDSATRYSYPAYDTTATTKSDALSGVTTS 120

436 YVSVMGDQGSGNISGGRYSIDALIRGGYHNNPDSATRYSYPAYDTTATTKSDALSSVTTS 120

1070 YVSVMGDQGSGNISGGRYSIDALIRGGYHNNPDSATRYSYPAYDTTATTKSDALSGVTTS 120

92 YVSVMGDQGSGNISGGRYSIDALIRGGYHNNPDSATRYSYPAYDTTATTKSDALSGVTTS 120

449 YVSVMGDQGSGNISGGRYSIDALIRGGYHNNPDSATRYSYPAYDTTATTKSDALSGVTTS 120

247 YVSVMGDQGSGNISGGRYSIDALIRGGYHNNPDSATRYSYPAYDTTATTKSDALSGVTTS 120

88-FA1090 YVSVMGDQGSGNISGGRYSIDALIRGGYHNNPDSATRYSYPAYDTTATTKSDALSGVTTS 120

90-AR205 YVSVMGDQGSGNISGGRYSIDALIRGGYHNNPDSATRYSYPAYDTTATTKSDALSGVTTS 120

460 YVSVMGDQGSGNISGGRYSIDALIRGGYHNNPDSATRYSYPAYDTTATTKSDALSGVTTS 120

2385 YVSVMGDQGSGNISGGRYSIDALIRGGYHNNPDSATRYSYPAYDTTATTKSDALSGVTTS 119

193-P9-17 YVSVMGDQGSGNISGGRYSIDALIRGGYHNNPDSATRYSYPAYDTTATTKSDALSGVTTS 119

458 YVSVMGDQGSGNISGGRYSIDALIRGGYHNNPDSATRYSYPAYDTTATTKSDALSGVTTS 119

65 YVSVMGDQGSGNISGGRYSIDALIRGGYQNNPESATQYSYPAYDTTATTKSDALSSVTTS 120

9 YVSVMGDQGSGNISGGRYSIDALIRGGYHNNPESATRYSYPAYDTTATTKSDALSSVTTS 120

2102 YVSVMGDQGSGNISGGRYSIDALIRGGYHNNPESATRYSYPTYDTTATTKADALSGVTSS 120

********** **************** :*.*:*: :** *:********:****.**:*

1467 TSLLNAPAAALTKNNGRKGERSAGLSVNGTGDYRNETLLANPRDVSFLTNLIQTVFYLRG 180

251 TSLLNAPAAALTKNNGRKGERSAGLSVNGTGDYRNETLLANPRDVSFLTNLIQTVFYLRG 180

475 TSLLNAPAAALTKNNGRKGERSAGLSVNGTGDYRNETLLANPRDVSFLTNLIQTVFYLRG 180

196 TSLLNAPAAALTKNNGRKGERSAGLSVNGTGDYRNETLLANPRDVSFLTNLIQTVFYLRG 180

1471 TSVLNAPAAALTKNNGRKGERSAGLSVNGTGDYRNETLLANPRDVSFLTNLIQTVFYLRG 180

464 TSVLNAPAAALTKNNGRKGERSAGLSVNGTGDYRNETLLANPRDVSFLTNLIQTVFYLRG 180

439 TSVLNAPAAALTKNNGRKGERSAGLSVNGTGDYRNETLLANPRDVSFLTNLIQTVFYLRG 180

446 TSVLNAPAAALTKNNGRKGERSAGLSVNGTGDYRNETLLANPRDVSFLTNLIQTVFYLRG 180

2501 TSVLNAPAAALTKNNGRKGERSAGLSVNGTGDYRNETLLANPRDVSFLTNLIQTVFYLRG 179

822 TSLLNAPAAALTKNNGRKGERSAGLSVNGTGDYRNETLLANPRDVSFLTNLIQTVFYLRG 179

472 TSVLNAPAAALTKNNGRKGERSAGLSVNGTGDYRNETLLANPRDVSFLTNLIQTVFYLRG 179

2657 TSVLNAPDAALTKNNGRKGERSAGLSVNGTGDYRNETLLANPRDVSFLTNLIQTVFYLRG 180

2369 TSVLNAPAAALTKNSGRKGERSAGLSVNGTGDYRNETLLANPRDVSFLTNLIQTVFYLRG 180

2143 TSVLNAPAAALTKNNGRKGERSAGLSVNGTGDYRNETLLANPRDVSFLTNLIQTVFYLRG 180

2665 TSVLNAPAAALTKNNGRKGERSAGLSVNGTGDYRNETLLANPRDVSFLTNLIQTVFYLRG 180

1048 TSVLNAPAAALTKNNGRKGERSAGLSVNGTGDYRNETLLANPRDVSFLTNLIQTVFYLRG 180

2477 TSVLNTPAAALTKNNGRKGERSAGLSVNGTGDYRNETLLANPRDVSFLTNLIQTVFYLRG 180

1047 TSVLNAPAAALTKNNGRKGERSAGLSVNGTGDYRNETLLANPRDVSFLTNLIQTVFYLRG 180

1238 TSVLNAPAAALTKNNGRKGERSAGLSVNGTGDYRNETLLANPRDVSFLTNLIQTVFYLRG 180

452 TSVLNAPAAALTKNNGRKGERSAGLSVNGTGDYRNETLLANPRDVSFLTNLIQTVFYLRG 180

456 TSVLNAPAAALTKNNGRKGERSAGLSVNGTGDYRNETLLANPRDVSFLTNLIQTVFYLRG 180

136 TSVLNAPAAALTKNNGRKGERSAGLSVNGTGDYRNETLLANPRDVSFLTNLIQTVFYLRG 180

2077 TSVLNAPAAALTKNNGRKGERSAGLSVNGTGDYRNETLLANPRDVSFLTNLIQTVFYLRG 180

1201 TSLLNAPAAALTKNSGHKGERSAGLSVNGTGDYRNETLLANPRDVSFLANLIQTVFYLRG 180

2176 TSLLNAPAAALTKNSGHKGERSAGLSVNGTDDYRNETLLANPRDVSFLTNLIQTVFYLRG 180

2175 TSLLNAPAAALTKNSGHKGERSAGLSVNGTDDYRNETLLANPRDVSFLTNLIQTVFYLRG 180

2702 TSVLNAPAAALTKNNGRKGERSAGLSVNGTGDYRNETLLANPRDVSFLTNLIQTVFYLRG 180

2653 TSLLNAPAAALTKNNGRKGERSAGLSVNGTDDYRNETLLANPRDVSFLTNLIQTVFYLRG 180

2604 TSVLNAPAAALTKNNGRKGERSAGLSVNGTGDYRNETLLANPRDVSFLTNLIQTVFYLRG 180

2603 TSVLNAPAAALTKNNGRKGERSAGLSVNGTDDYRNETLLANPRDVSFLTNLIQTVFYLRG 180

2599 TSVLNAPAAALTKNNGRKGERSAGLSVNGTGDYRNETLLANPRDVSFLTNLIQTVFYLRG 180

2496 TSVLNAPAAALTKNNGRKGERSAGLSVNGTDDYRNETLLANPRDVSFLTNLIQTVFYLRG 180

2375 TSVLNAPAAALTKNSGHKGERSAGLSVNGTGDYRNETLLANPRDVSFLTNLIQTVFYLRG 180

2347 TSVLNAPAAALTKNNGRKGERSAGLSVNGTGDYRNETLLANPRDVSFLTNLIQTVFYLRG 180

2170 TSVLNAPAAALTKNNGRKGERSAGLSVNGTGDYRNETLLVNPRDVSFLTNLIQTVFYLRG 180

2152 TSLLNAPAAALTKNNGRKGERSAGLSVNGTGDYRNETLLANPRDVSFLTNLIQTVFYLRG 180

2083 TSVLNAPAAALTKNNGRKGERSAGLSVNGTGDYRNETLLANPRDVSFLTNLIQTVFYLRG 180

1498 TSVLNAPAAALTKNNGRKGERSAGLSVNGTGDYRNETLLANPRDVSFLTNLIQTVFYLRG 180

1481 TSVLNAPAAALTKNNGRKGERSAGLSVNGTGDYRNETLLANPRDVSFLTNLIQTVFYLRG 180

1468 TSVLNAPAAALTKNNGRKGERSAGLSVNGTGDYRNETLLANPRDVSFLTNLIQTVFYLRG 180

1459 TSVLNAPAAALTKNNGRKGERSAGLSVNGTGDYRNETLLANPRDVSFLTNLIQTVFYLRG 180

1412 TSVLNAPAAALTKNNGRKGERSAGLSVNGTGDYRNETLLANPRDVSFLTNLIQTVFYLRG 180

1296 TSVLNAPAAALTKNNGRKGERSAGLSVNGTGDYRNETLLANPRDVSFLTNLIQTVFYLRG 180

1295 TSLLNAPAAALTKNNGRKGERSAGLSVNGTGDYRNETLLANPRDVSFLTNLIQTVFYLRG 180

1202 TSVLNAPAAALTKNSGRKGERSAGLSVNGTGDYRNETLLANPRDVSFLTNLIQTVFYLRG 180

1125 TSVLNAPAAALTKNSGHKGERSAGLSVNGTDDYRNETLLANPRDVSFLTNLIQTVFYLRG 180

1057 TSLLNAPAAALTKNNGRKGERSAGLSVNGTGDYRNETLLANPRDVSFLTNLIQTVFYLRG 180

678 TSVLNAPAAALTKNNGRKGERSAGLSVNGTGDYRNETLLANPRDVSFLTNLIQTVFHLRG 180

582 TSVLNAPAAALTKNNGRKGERSAGLSVNGTDDYRNETLLANPRDVSFLTNLIQTVFYLRG 180

469 TSVLNAPAAALTKNNGRKGERSAGLSVNGTGDYRNETLLANPRDVSFLTNLIQTVFYLRG 180

466 TSLLNAPAAALTKNNGRKGERSAGLSVNGTGDYRNETLLANPRDVSFLTNLIQTVFYLRG 180

462 TSLLNAPAAALTKNNGRKGERSAGLSVNGTGDYRNETLLANPRDVSFLTNLIQTVFYLRG 180

459 TSVLNAPAAALTKNNGRKGERSAGLSVNGTGDYRNETLLANPRDVSFLTNLIQTVFYLRG 180

437 TSLLNAPAAALTKNNGRKGERSAGLSVNGTGDYRNETLLANPRDVSFLTNLIQTVFYLRG 180

431 TSLLNAPAAALTKNNGRKGERSAGLSVNGTGDYRNETLLANPRDVSFLTNLIQTVFYLRG 180

2367 TSLLNAPAAALTKNNGRKGERSAGLSVNGTGDYRNETLLANPRDVSFLTNLIQTVFYLRG 180

2326 TSVLNAPAAALTKNSGHKGERSAGLSVNGTDDYRNETLLANPRDVSFLTNLIQTVFYLRG 180

2283 TSLLNAPAAALTKNNGRKGERSAGLSVNGTGDYRNETLLANPRDVSFLTNLIQTVFYLRG 180

2282 TSLLNAPAAALTKNNGCKGERSAGLSVNGTGDYRNETLLANPRDVSFLTNLIQTVFYLRG 180

1456 TSLLNAPAAALTKNNGRKGERSAGLSVNGTGDYRNETLLANPRDVSFLTNLIQTVFYLRG 180

1242 TSVLNAPAAALTKNNGRKGERSAGLSVNGTDDYRNETLLANPRDVSFLTNLIQTVFYLRG 180

1055 TSLLNAPAAALTKNNGRKGERSAGLSVNGTGDYRNETLLANPRDVSFLTNLIQTVFYLRG 180

1054 TSVLNAPAAALTKNSGRKGERSAGLSVNGTDDYRNETLLANPRDVSFLTNLIQTVFYLRG 180

789 TSLLNAPAAALTKNNGRKGERSAGLSVNGTGDYRNETLLANPRDVSFLTNLIQTVFYLRG 180

600 TSLLNAPAAALTKNNGRKGERSAGLSVNGTGDYRNETLLANPRDVSFLTNLIQTVFYLRG 180

455 ASLLNAPAAALTKNNGRKGERSAGLSVNGTGDYRNETLLANPRDVSFLTNLIQTVFYLRG 180

451 TSVLNAPAAALTKNNGRKGERSAGLSVNGTGDYRNETLLANPRDVSFLTNLIQTVFYLRG 180

2337 TSLLNAPAAALTKNNGRKGERSAGLSVNGTGDYRNETLLANPRDVSFLTNLIQTVFYLRG 180

2089 TSLLNAPAAALTKNNGRKGERSAGLSVNGTGDYRNETLLANPRDVSFLTNLIQTVFYLRG 180

468 TSLLNAPAAALTKNNGRKGERSAGLSVNGTGDYRNETLLANPRDVSFLTNLIQTVFYLRG 180

442 TSVLNAPAAALTKNSGHKGERSAGLSVNGTDDYRNETLLANPRDVSFLTNLIQTVFYLRG 180

2040 TSVLNAPAAALTKNNGRKGERSAGLSVNGTGDYRNETLLANPRDVSFLTNLIQTVFYLRG 180

1075 TSLLNAPAAALTKNNGRKGERSAGLSVNGTGDYRNETLLANPRDVSFLTNLIQTVFYLRG 180

521 TSVLNAPAAALTKNNGRKGERSAGLSVNGTGDYRNETLLANPRDVSFLTNLIQTVFYLRG 180

2305 TSLLNAPAAALTKNNGRKGERSAGLSVNGTGDYRNETLLANPRDVSFLTNLIQTVFYLRG 180

1473 TSVLNAPAAALTKNSGRKGERSAGLSVNGTDDYRNETLLANPRDVSFLTNLIQTVFYLRG 180

1284 TSLLNAPAAALTKNNGRKGERSAGLSVNGTGDYRNETLLANPRDVSFLTNLIQTVFYLRG 180

679 TSLLNAPAAALTKNNGRKGERSAGLSVNGTGDYRNETLLANPRDVSFLTNLIQTVFYLRG 180

2146 TSLLNAPAAALTKNNGRKGERSAGLSVNGTGDYRNETLLANPRDVSFLTNLIQTVFYLRG 180

1046 TSVLNAPAAALTKNNGRKGERSAGLSVNGTGDYRNETLLANPRDVSFLTNLIQTVFYLRG 180

605 TSVLNAPAAALTKNNGRKGERSAGLSVNGTGDYRNETLLANPRDVSFLTNLIQTVFYLRG 180

1053 TSVLNAPAAALTKNNGRKGERSAGLSVNGTGDYRNETLLANPRDVSFLTNLIQTVFYLRG 180

855 TSLLNAPAAALTKNNGRKGERSAGLSVNGTGDYRNETLLANPRDVSFLTNLIQTVFYLRG 180

1388 TSVLNAPAAALTKNNGRKGERSAGLSVNGTGDYRNETLLANPRDVSFLTNLIQTVFYLRG 180

362 TSVLNAPAAALTKNNGRKGKRSAGLSVNGTGDYRNETLLANPRDVSFLTNLIQTVFYLRG 180

438 TSLLNAPAAALTKNNGRKGERSAGLSVNGTGDYRNETLLANPRDVSFLTNLIQTVFYLRG 180

1017 TSLLNAPAAALTKNNGRKGERSAGLSVNGTGDYRNETLLANPRDVSFLTNLIQTVFYLRG 180

436 TSLLNAPAAALTKNNGRKGERSAGLSVNGTGDYRNETLLANPRDVSFLTNLIQTVFYLRG 180

1070 TSVLNAPAAALTKNNGRKGERSAGLSVNGTGDYRNETLLANPRDVSFLTNLIQTVFYLRG 180

92 TSVLNAPAAALTKNNGRKGERSAGLSVNGTDDYRNETLLANPRDVSFLTNLIQTVFYLRG 180

449 TSLLNAPAAALTKNNGRKGERSAGLSVNGTGDYRNETLLANPRDVSFLTNLIQTVFYLRG 180

247 TSVLNAPAAALTKNNGRKGERSAGLSVNGTGDYRNETLLANPRDVSFLTNLIQTVFYLRG 180

88-FA1090 TSLLNAPAAALTKNNGRKGERSAGLSVNGTGDYRNETLLANPRDVSFLTNLIQTVFYLRG 180

90-AR205 TSVLNAPAAALTKNNGRKGERSAGLSVNGTGDYRNETLLANPRDVSFLTNLIQTVFYLRG 180

460 TSVLNAPAAALTKNNGRKGERSAGLSVNGTGDYRNETLLANPRDVSFLTNLIQTVFYLRG 180

2385 TSVLNAPAAALTKNNGRKGERSAGLSVNGTGDYRNETLLANPRDVSFLTNLIQTVFYLRG 179

193-P9-17 TSLLNAPAAALTKNSGHKGERSAGLSVNGTDDYRNETLLANPRDVSFLTNLIQTVFYLRG 179

458 TSLLNAPAAALTKNSGHKGERSAGLSVNGTDDYRNETLLANPRDVSFLTNLIQTVFYLRG 179

65 TSLLNAPAAALTRNSGRKGERSAGLSVNGTGDYRNETLLANPRDVSFLTNLIQTVFYLRG 180

9 TSLLNAPAAALTRNSGRKGERSAGLSVNGMGDYRNETLLANPRDVSFLTNLIQTVFYLRG 180

2102 TSLLNAPAAALTRNSGRKGERSAGLSVNGTGDYRNETLLANPRDVSFLTNLIQTVFYLRG 180

:*:**:* ****:*.* **:********* .********.********:*******:***

1467 IEVVPPEYADTDVFVTVDVFGTVRSRTELHLYNAETLKAQTKLEYFAVDRDSRKLLIAPK 240

251 IEVVPPEYADTDVFVTVDVFGTVRSRTELHLYNAETLKAQTKLEYFAVDRDSRKLLIAPK 240

475 IEVVPPEYADTDVFVTVDVFGTVRSRTELHLYNAETLKAQTKLEYFAVDRDSRKLLIAPK 240

196 IEVVPPEYADTDVFVTVDVFGTVRSRTELHLYNAETLKAQTKLEYFAVDRDSRKLLIAPK 240

1471 IEVVPPEYADTDVFVTVDVFGTVRSRTELHLYNAETLKAQTKLEYFAVDRDSRKLLIAPK 240

464 IEVVPPEYADTDVFVTVDVFGTVRSRTELHLYNAETLKAQTKLEYFAVDRDSRKLLIAPK 240

439 IEVVPPEYADTDVFVTVDVFGTVRSRTELHLYNAETLKAQTKLEYFAVDRDSRKLLIAPK 240

446 IEVVPPEYADTDVFVTVDVFGTVRSRTELHLYNAETLKAQTKLEYFAVDRDSRKLLIAPK 240

2501 IEVVPPEYADTDVFVTVDVFGTVRSRTELHLYNAETLKAQTKLEYFAVDRDSRKLLIAPK 239

822 IEVVPPEYADTDVFVTVDVFGTVRSRTELHLYNAETLKAQTKLEYFAVDRDSRKLLIAPK 239

472 IEVVPPEYADTDVFVTVDVFGTVRSRTELHLYNAETLKAQTKLEYFAVDRDSRKLLIAPK 239

2657 IEVVPPEYADTDVFVTVDVFGTVRSRTELHLYNAETLKAQTKLEYFAVDRDSRKLLIAPK 240

2369 IEVVPPEYADTDVFVTVDVFGTVRSRTELHLYNAETLKAQTKLEYFAVDRDSRKLLIAPK 240

2143 IEVVPPEYADTDVFVTVDVFGTVRSRTELHLYNAETLKAQTKLEYFAVDRDSRKLLIAPK 240

2665 IEVVPPEYADTDVFVTVDVFGTVRSRTELHLYNAETLKAQTKLEYFAVDRDSRKLLIAPK 240

1048 IEVVPPEYADTDVFVTVDVFGTVRSRTELHLYNAETLKAQTKLEYFAVDRDSRKLLIAPK 240

2477 IEVVPPEYADTDVFVTVDVFGTVRSRTELHLYNAETLKAQTKLEYFAVDRDSRKLLIAPK 240

1047 IEVVPPEYADTDVFVTVDVFGTVRSRTELHLYNAETLKAQTKLEYFAVDRDSRKLLIAPK 240

1238 IEVVPPEYADTDVFVTVDVFGTVRSRTELHLYNAETLKAQTKLEYFAVDRDSRKLLIAPK 240

452 IEVVPPEYADTDVFVTVDVFGTVRSRTELHLYNAETLKAQTKLEYFAVDRDSRKLLIAPK 240

456 IEVVPPEYADTDVFVTVDVFGTVRSRTELHLYNAETLKAQTKLEYFAVDRDSRKLLIAPK 240

136 IEVVPPEYADTDVFVTVDVFGTVRSRTELHLYNAETLKAQTKLEYFAVDRDSRKLLIAPK 240

2077 IEVVPPEYADTDVFVTVDVFGTVRSRTELHLYNAETLKAQTKLEYFAVDRDSRKLLIAPK 240

1201 IEVVPPEYADTDVFVTVDVFGTVRSRTELHLYNAETLKAQTKLEYFAVDRDSRKLLIAPK 240

2176 IEVVPPEYADTDVFVTVDVFGTVRSRTELHLYNAETLKAQTKLEYFAVDRDSRKLLIAPK 240

2175 IEVVPPEYADTDVFVTVDVFGTVRSRTELHLYNAETLKAQTKLEYFAVDRDSRKLLIAPK 240

2702 IEVVPPEYADTDVFVTVDVFGTVRSRTELHLYNAETLKAQTKLEYFAVDRDSRKLLIAPK 240

2653 IEVVPPEYADTDVFVTVDVFGTVRSRTELHLYNAETLKAQTKLEYFAVDRDSRKLLIAPK 240

2604 IEVVPPEYADTDVFVTVDVFGTVRSRTELHLYNAETLKAQTKLEYFAVDRDSRKLLIAPK 240

2603 IEVVPPEYADTDVFVTVDVFGTVRSRTELHLYNAETLKAQTKLEYFAVDRDSRKLLIAPK 240

2599 IEVVPPEYADTDVFVTVDVFGTVRSRTELHLYNAETLKAQTKLEYFAVDRDSRKLLIAPK 240

2496 IEVVPPEYADTDVFVTVDVFGTVRSRTELHLYNAETLKAQTKLEYFAVDRDSRKLLIAPK 240

2375 IEVVPPEYADTDVFVTVDVFGTVRSRTELHLYNAETLKAQTKLEYFAVDRDSRKLLIAPK 240

2347 IEVVPPEYADTDVFVTVDVFGTVRSRTELHLYNAETLKAQTKLEYFAVDRDSRKLLIAPK 240

2170 IEVVPPEYADTDVFVTVDVFGTVRSRTELHLYNAETLKAQTKLEYFAVDRDSRKLLIAPK 240

2152 IEVVPPEYADTDVFVTVDVFGTVRSRTELHLYNAETLKAQTKLEYFAVDRDSRKLLIAPK 240

2083 IEVVPPEYADTDVFVTVDVFGTVRSRTELHLYNAETLKAQTKLEYFAVDRDSRKLLIAPK 240

1498 IEVVPPEYADTDVFVTVDVFGTVRSRTELHLYNAETLKAQTKLEYFAVDRDSRKLLIAPK 240

1481 IEVVPPEYADTDVFVTVDVFGTVRSRTELHLYNAETLKAQTKLEYFAVDRDSRKLLIAPK 240

1468 IEVVPPEYADTDVFVTVDVFGTVRSRTELHLYNAETLKAQTKLEYFAVDRNSRKLLIAPK 240

1459 IEVVPPEYADTDVFVTVDVFGTVRSRTELHLYNPETLKAQTKLEYFAVDRDSRKLLIAPK 240

1412 IEVVPPEYADTDVFVTVDVFGTVRSRTELHLYNAETLKAQTKLEYFAVDRDSRKLLIAPK 240

1296 IEVVPPEYADTDVFVTVDVFGTVRSRTELHLYNAETLKAQTKLEYFAVDRDSRKLLIAPK 240

1295 IEVVPPEYADTDVFVTVDVFGTVRSRTELHLYNAETLKAQTKLEYFAVDRDSRKLLIAPK 240

1202 IEVVPPEYADTDVFVTVDVFGTVRSRTELHLYNAETLKAQTKLEYFAVDRDSRKLLIAPK 240

1125 IEVVPPEYADTDVFVTVDVFGTVRSRTELHLYNAETLKAQTKLEYFAVDRDSRKLLIAPK 240

1057 IEVVPPEYADTDVFVTVDVFGTVRSRTELHLYNAETLKAQTKLEYFAVDRDSRKLLIAPK 240

678 IEVVPPEYADTDVFVTVDVFGTVRSRTELHLYNAETLKAQTKLEYFAVDRDSRKLLIAPK 240

582 IEVVPPEYADTDVFVTVDVFCTVRSRTELHLYNAETLKAQTKLEYFAVDRDSRKLLIAPK 240

469 IEVVPPEYADTDVFVTVDVFGTVRSRTELHLYNAETLKAQTKLEYFAVDRDSRKLLIAPK 240

466 IEVVPPEYADTDVFVTVDVFGTVRSRTELHLYNAETLKAQTKLEYFAVDRDSRKLLIAPK 240

462 IEVVPPEYADTDVFVTVDVFGTVRSRTELHLYNAETLKAQTKLEYFAVDRDSRKLLIAPK 240

459 IEVVPPEYADTDVFVTVDVFGTVRSRTELHLYNAETLKAQTKLEYFAVDRDSRKLLIAPK 240

437 IEVVPPEYADTDVFVTVDVFGTVRSRTELHLYNAETLKAQTKLEYFAVDRDSRKLLIAPK 240

431 IEVVPPEYADTDVFVTVDVFGTVRSRTELHLYNAETLKAQTKLEYFAVDRDSRKLLIAPK 240

2367 IEVVPPEYADTDVFVTVDVFGTVRSRTELHLYNAKTLKAQTKPEYFAVDRDSRKLLIAPK 240

2326 IEVVPPEYADTDVFVTVDVFGTVRSRTELHLYNAETLKAQTKLEYFAVDRDSRKLLIAPK 240

2283 IEVVPPEYADTDVFVTVDVFGTVRSRTELHLYNAETLKAQTKLEYFAVDRDSRKLLIAPK 240

2282 IEVVPPEYADTDVFVTVDVFGTVRSRTELHLYNAETLKAQTKLEYFAVDRDSRKLLIAPK 240

1456 IEVVPPEYADTDVFVTVDVFGTVRSRTELHLYNAETLKAQTKLEYFAVDRDSRKLLIAPK 240

1242 IEVVPPEYADTDVFVTVDVFGTVRSRTELHLYNAETLKAQTKLEYFAVDRDSRKLLIAPK 240

1055 IEVVPPEYADTDVFVTVDVFGTVRSRTELHLYNAETLKAQTKLEYFAVDRDSRKLLIAPK 240

1054 IEVVPPEYADTDVFVTVDVFGTVRSRTELHLYNAETLKAQTKLEYFAVDRDSRKLLIAPK 240

789 IEVVPPEYADTDVFVTVDVFDTVRSRTELHLYNAETLKAQTKLEYFAVDRDSRKLLIAPK 240

600 IEVVPPEYADTDVFVTVDVFGTVRSRTELHLYNAETLKAQTKLEYFAVDRDSRKLLIAPK 240

455 IEVVPPEYADTDVFVTVDVFGTVRSRTELHLYNAETLKAQTKLEYFAVDRDSRKLLIAPK 240

451 IEVVPPEYADTDVFVTVDVFGTVRSRTELHLYNAETLKAQTKLEYFAVDRDSRKLLIAPK 240

2337 IEVVPPEYADTDVFVTVDVFGTVRSRTELHLYNAETLKAQTKLEYFAVDRDSRKLLIAPK 240

2089 IEVVPPEYADTDVFVTVDVFGTVRSRTELHLYNAETLKAQTKLEYFAVDRDSRKLLIAPK 240

468 IEVVPPEYADTDVFVTVDVFGTVRSRTELHLYNAETLKAQTKLEYFAVDRDSRKLLIAPK 240

442 IEVVPPEYADTDVFVTVDVFGTVRSRTELHLYNAETLKAQTKLEYFAVDRDSRKLLIAPK 240

2040 IEVVPPEYADTDVFVTVDVFGTVRSRTELHLYNAKTLKAQTKLEYFAVDRDSRKLLIAPK 240

1075 IEVVPPEYADTDVFVTVDVFGTVRSRTELHLYNAETLKAQTKLEYFAVDRDSRKLLIAPK 240

521 IEVVPPEYADTDVFVTVDVFGTVRSRTELHLYNAETLKAQTKLEYFAVDRDSRKLLIAPK 240

2305 IEVVPPEYADTDVFVTVDVFGIVRSRTELHLYNAETLKAQTKLEYFAVDRDSRKLLIAPK 240

1473 IEVVPPEYADTDVFVTVDVFGTVRSRTELHLYNAETLKAQTKLEYFAVDRDSRKLLIAPK 240

1284 IEVVPPEYADTDVFVTVDVFGTVRSRTELHLYNAETLKAQTKLEYFAVDRDSRKLLIAPK 240

679 IEVVPPEYADTDVFVTVDVFGTVRSRTELHLYNAETLKAQTKLEYFAVDRDSRKLLIAPK 240

2146 IEVVPPEYADTDVFVTVDVFDTVRSRTELHLYNAETLKAQTKLEYFAVDRDSRKLLIAPK 240

1046 IEVVPPEYADTDVFVTVDVFGTVRSRTELHLYNAETLKAQTKLEYFAVDRDSRKLLIAPK 240

605 IEVVPPEYADTDVFVTVDVFGTVRSRTELHLYNAETLKAQTKLEYFAVDRDSRKLLIAPK 240

1053 IEVVPPEYADTDVFVTVDVFGTVRSRTELHLYNAETLKAQTKLEYFAVDRDSRKLLIAPK 240

855 IEVVPPEYADTDVFVTVDVFGTVRSRTELHLYNAETLKAQTKLEYFAVDRDSRKLLIAPK 240

1388 IEVVPPEYADTDVFVTVDVFGTVRSRTELHLYNAETLKAQTKLEYFAVDRDSRKLLIAPK 240

362 IEVVPPEYADTDVFVTVDVFGTVRSRTELHLYNAETLKAQTKLEYFAVDRDSRKLLIAPK 240

438 IEVVPPEYADTDVFVTVDVFGTVRSRTELHLYNAETLKAQTKLEYFAVDRDSRKLLIAPK 240

1017 IEVVPPEYADTDVFVTVDVFGTVRSRTELHLYNAKTLKAQTKLEYFAVDRDSRKLLIAPK 240

436 IEVVPPEYADTDVFVTVDVFGTVRSRTELHLYNAETLKAQTKLEYFAVDRDSRKLLIAPK 240

1070 IEVVPPEYADTDVFVTVDVFGTVRSRTELHLYNAETLKAQTKLEYFAVDRDSRKLLIAPK 240

92 IEVVPPEYADTDVFVTVDVFGTVRSRTELHLYNAETLKAQTKLEYFAVDRDSRKLLIAPK 240

449 IEVVPPEYADTDVFVTVDVFGTVRSRTELHLYNAETLKAQTKLEYFAVDRDSRKLLIAPK 240

247 IEVVPPEYADTDVFVTVDVFGTVRSRTELHLYNAETLKAQTKLEYFAVDRDSRKLLIAPK 240

88-FA1090 IEVVPPEYADTDVFVTVDVFGTVRSRTELHLYNAETLKAQTKLEYFAVDRDSRKLLIAPK 240

90-AR205 IEVVPPEYADTDVFVTVDVFGTVRSRTELHLYNAETLKAQTKLEYFAVDRDSRKLLIAPK 240

460 IEVVPPEYADTDVFVTVDVFGTVRSRTELHLYNAETLKAQTKLEYFAVDRDSRKLLIAPK 240

2385 IEVVPPEYADTDVFVTVDVFGTVRSRTELHLYNAETLKAQTKLEYFAVDRDSRKLLIAPK 239

193-P9-17 IEVVPPEYADTDVFVTVDVFGTVRSRTELHLYNAETLKAQTKLEYFAVDRDSRKLLIAPK 239

458 IEVVPPEYADTDVFVTVDVFGTVRSRTELHLYNAETLKAQTKLEYFAVDRDSRKLLIAPK 239

65 IEVVPPEYADTDVFVTVDVFGTVRSRTELHLYNAETLKAQTKLEYFAVDRDSRKLLIAPK 240

9 IEVVPPEYADTDVFVTVDVFGTVRSRTELHLYNAETLKAQTKLEYFAVDRDSRKLLIAPK 240

2102 IEVVPPEYADTDVFVTVDVFGTVRSRTELHLYNAETLKAQTKLEYFAVDRDSRKLLIAPK 240

******************** *********** :******* *******:*********

1467 TAAYESQYQEQYALWMGPYSVGKTVKASDRLMVDFSDITPYGDTTAQNRPDFKQNNGKNP 300

251 TAAYESQYQEQYALWMGPYSVGKTVKASDRLTVDFSDITPYGDTTAQNRPDFKQNNGKNP 300

475 TAAYESQYQEQYALWMGPYSVGKTVKASDRLTVDFSDITPYGDTTAQNRPDFKQNNGKNP 300

196 TAAYESQYQEQYALWMGPYSVGKTVKASDRLMVDFSDITPYGDTTAQNRPDFKQNNGKNP 300

1471 TAAYESQYQEQYALWMGPYSVGKTVKASDCLMVDFSDITPYGDTTAQNRPDFKQNNGKNP 300

464 TAAYESQYQEQYALWMGPYSVGKTVKASDRLTVDFSDITPYGDTTAQNRPDFKQNNGKNP 300

439 TAAYESQYQEQYALWMGPYSVGKTVKASDRLMVDFSDITPYGDTTAQNRPDFKQNNGKNP 300

446 TAAYESQYQEQYALWMGPYSVGKTVKASDRLMVDFSDITPYGDTTAQNRPDFKQNNGKNP 300

2501 TAAYESQYQEQYALWMGPYSVGKTVKASDRLMVDFSDITPYGDTTAQNRPDFKQNNGKNP 299

822 TAAYESQYQEQYALWMGPYSVGKTVKASDRLMVDFSDITPYGDTTAQNRPDFKQNNGKNP 299

472 TAAYESQYQEQYALWMGPYSVGKTVKASDRLMVDFSDITPYGDTTAQNRPDFKQNNGKNP 299

2657 TAAYESQYQEQYALWMGPYSVGKTVKASDRLMVDFSDITPYGDTTAQNRPDFKQNNGKNP 300

2369 TAAYESQYQEQYALWMGPYSVGKTVKASDRLMVDFSDITPYGDTTAQNRPDFKQNNGKNP 300

2143 TAAYESQYQEQYALWMGPYSVGKTVKASDRLMVDFSDITPYGDTTAQNRPDFKQNNGKNP 300

2665 TAAYESQYQEQYALWMGPYSVGKTVKASDRLTVDFSDITPYGDTTAQNRPDFKQNNGKNP 300

1048 TAAYESQYQEQYALWMGPYSVGKTVKASDRLMVDFSDITPYGDTTAQNRPDFKQNNGKNP 300

2477 TAAYESQYQEQYALWMGPYSVGKTVKASDRLMVDFSDITPYGDTTAQNRPDFKQNNGKNP 300

1047 TAAYESQYQEQYALWMGPYSVGKTVKASDRLMVDFSDITPYGDTTAQNRPDFKQNNGKNP 300

1238 TAAYESQYQEQYALWMGPYSVGKTVKASDRLMVDFSDITAYGDTTAQNRPDFKQNNGKNP 300

452 TAAYESQYQEQYALWMGPYSVGKTVKASDRLMVDFSDITPYGDTTAQNRPDFKQNNGKNP 300

456 TAAYESQYQEQYALWMGPYSVGKTVKASDRLMVDFSDITAYGDTTAQNRPDFKQNNGKNP 300

136 TAAYESQYQEQYALWMGPYSVGKTVKASDRLMVDFSDITPYGDTTAQNRPDFKQNNGKNP 300

2077 TAAYESQYQEQYALWMGPYSVGKTVKASDRLMVDFSDITPYGDTTAQNRPDFKQNNGKNP 300

1201 TAAYESQYQEQYALWMGPYSVGKTVKASDRLMVDFSDITPYGDTTAQNRPDFKQNNGKNP 300

2176 TAAYESQYQEQYALWMGPYSVGKTVKASDRLMVDFSDITPYGDTTAQNRPDFKQNNGKNP 300

2175 TAAYESQYQEQYALWMGPYSVGKTVKASDRLMVDFSDITPYGDTTAQNRPDFKQNNGKNP 300

2702 TAAYESQYQEQYALWMGPYSVGKTVKASDRLMVDFSDITPYGDTTAQNRPDFKQNNGKNP 300

2653 TAAYESQYQEQYALWMGPYSVGKTVKASDRLMVDFSDITPYGDTTAQNRPDFKQNNGKNP 300

2604 TAAYESQYQEQYALWMEPYSVGKTVKASDRLMVDFSDITPYGDTTAQNRPDFKQNNGKNP 300

2603 TAAYESQYQEQYALWMGPYSVGKTVKASDRLMVDFSDITPYGDTTAQNRPDFKQNNGKNP 300

2599 TVAYESQYQEQYALWMGPYSVGKTVKASDRLMVDFSDITPYGDTTAQNRPDFKQNNGKNP 300

2496 TAAYESQYQEQYALWMGPYSVGKTVKASDRLMVDFSDITPYGDTTAQNRPDFKQNNGKNP 300

2375 TAAYESQYQEQYALWMGPYSVGKTVKASDRLMVDFSDITPYGDTTAQNRPDFKQNNGKNP 300

2347 TAAYESQYQEQYALWMGPYSVGKTVKASDRLMVDFSDITPYGDTTAQNRPDFKQNNGKNP 300

2170 TAAYESQYQEQYALWMGPYSVGKTVKASDRLMVDFSDITPYGDTTAQNRPDFKQNNGKNP 300

2152 TAAYESQYQEQYALWMGPYSVGKTVKASDRLMVDFSDITPYGDTTAQNRPDFKQNNGKNP 300

2083 TAAYESQYQEQYALWMRPYSVGKTVKASDRLMVDFSDITPYGDTTAQNRPDFKQNNGKNP 300

1498 TAAYESQYQEQYALWMGPYSVGKTVKASDRLMVDFSDITPYGDTTAQNRPDFKQNNGKNP 300

1481 TAAYESQYQEQYALWMGPYSVGKTVKASDRLMVDFSDITPYGDTTAQNRPDFKQNNGKNP 300

1468 TAAYESQYQEQYALWMGPYSVGKTVKASDRLMVDFSDITPYGDTTAQNRPDFKQNNGKNP 300

1459 TAAYESQYQEQYALWMGPYSVGKTVKASDRLMVDFSDITPYGDTTAQNRPDFKQNNGKNP 300

1412 TAAYESQYQEQYALWMGPYSVGKTVKASDRLMVDFSDITPYGDTTAQNRPDFKQNNGKNP 300

1296 TAAYESQYQEQYALWMGPYSVGKTVKASDRLMVDFSDITPYGDTTAQNRPDFKQNNGKNP 300

1295 TAAYESQYQEQYALWMGPYSVGKTVKASDRLMVDFSDITPYGDTTAQNRPDFKQNNGKNP 300

1202 TAAYESQYQEQYALWMGPYSVGKTVKASDRLMVDFSDITPYGDTTAQNRPDFKQNNGKNP 300

1125 TAAYESQYQEQYALWMGPYSVGKTVKASDRLMVDFSDITAYGDTTAQNRPDFKQNNGKNP 300

1057 TAAYESQYQEQYALWMGPYSVGKTVKASDRLMVDFSDITPYGDTTAQNRPDFKQNNGKNP 300

678 TAAYESQYQEQYALWMGPYSVGKTVKASDRLMVDFSDITPYGDTTAQNRPDFKQNNGKNP 300

582 TAAYESQYQEQYALWMGPYSVGKTVKASDRLMVDFSDITPYGDTTAQNRPDFKQNNGKNP 300

469 TATYESQYQEQYALWMGPYSVGKTVKASDRLMVDFSDITPYGDTTAQNRPDFKQNNGKNP 300

466 TAAYESQYQEQYALWMGPYSVGKTVKASDRLMVDFSDITPYGDTTAQNRPDFKQNNGKNP 300

462 TAAYESQYQEQYALWMGPYSVGKTVKASDRLMVDFSDITPYGDTTAQNRPDFKQNNGKNP 300

459 TAAYESQYQEQYALWMGPYSVGKTVKASDRLMVDFSDITPYGDTTAQNRPDFKQNNGKNP 300

437 TAAYESQYQEQYALWMGPYSVGKTVKASDRLMVDFSDITPYGDTTAQNRPDFKQNNGKNP 300

431 TAAYESQYQEQYALWMGPYSVGKTVKASDRLMVDFSDITPYGDTTAQNRPDFKQNNGKNP 300

2367 TAAYESQYQEQYALWMGPYSVGKTVKASDRLMVDFSDITPYGDTTAQNRPDFKQNNGKNP 300

2326 TAAYESQYQEQYALWMGPYSVGKTVKASDRLMVDFSDITPYGDTTAQNRPDFKQNNGKNP 300

2283 TAAYESQYQEQYALWMGPYSVGKTVKASDRLMVDFSDITAYGDTTAQNRPDFKQNNGKNP 300

2282 TAAYESQYQEQYALWMGPYSVGKTVKASDRLMVDFSDITPYGDTTAQNRPDFKQNNGKNP 300

1456 TAAYESQYQEQYALWMGPYSVGKTVKASDRLMVDFSDITPYGDTTAQNRPDFKQNNGKNS 300

1242 TAAYESQYQEQYALWMGPYSVGKTVKASDRLMVDFSDITPYGDTTAQNRPDFKQNNGKNP 300

1055 TAAYESQYQEQYALWMGPYSVGKTVKASDRLMVDFSDITPYGDTTAQNRPDFKQNNGKNP 300

1054 TAAYESQYQEQYALWMGPYSVGKTVKASDRLMVDFSDITPYGDTTAQNRPDFKQNNGKNP 300

789 TAAYESQYQEQYALWIGPYSVGKTVKASDRLMVDFSDITPYGDTTAQNRPDFKQNNGKNP 300

600 TAAYESQYQEQYALWMRPYSVGKTVKASDRLMVDFSDITPYGDTTAQNRPDFKQNNGKNP 300

455 TAAYESQYQEQYALWMGPYSVGKTVKASDRLMVDFSDITPYGDTTAQNRPDFKQNNGKNP 300

451 TAAYESQYQGQYALWMGPYSVGKTVKASDRLMVDFSDITPYGDTTAQNRPDFKQNNGKNP 300

2337 TAAYESQYQEQYALWMRPYSVGKTVKASDRLMVDFSDITPYGDTTAQNRPDFKQNNGKNP 300

2089 TAAYESQYQEQYALWMGPYSVGKTVKASDRLMVDFSDITPYGDTTAQNRPDFKQNNGKNP 300

468 TAAYESQYQEQYALWMGPYSVGKTVKALDRLMVDFSDITPYGDTTAQNRPDFKQNNGKNP 300

442 TAAYESQYQEQYALWMGPYSVGKTVKASDRLMVDFSDITPYGDTTAQNRPDFKQNNGKNP 300

2040 TAAYESQYQEQYALWMGPYSVGKTVKASDRLMVDFSDITPYGDTTAQNRPDFKQNNGKNP 300

1075 TAAYESQYQEQYALWMGPYSVGKTVKASDRLTVDFSDITPYGDTTAQNRPDFKQNNGKNP 300

521 TAAYESQYQEQYALWMGPYSVGKTVKASDRLMVDFSDITPYGDTTAQNRPDFKQNNGKNP 300

2305 TAAYESQYQEQYALWMGPYSVGKTVKASDRLMVDFSDITPYGDTTAQNRPDFKQNNGKNP 300

1473 TAAYESQYQEQYALWMGPYSVGKTVKASDRLMVDFSDITPYGDTTAQNRPDFKQNNGKNP 300

1284 TAAYESQYQEQYALWMGPYSVGKTVKASDRLMVDFSDITPYGDTTAQNRPDFKQNNGKNP 300

679 TAAYESQYQEQYALWMGPYSVGKTVKASDRLMVDFSDITPYGDTTAQNRPDFKQNNGKNP 300

2146 TAAYESQYQEQYALWMGPYSVGKTVKASDRLMVDFSDITPYGDTTAQNRPDFKQNNGKNP 300

1046 TAAYESQYQEQYALWMGPYSVGKTVKASDRLMVDFSDITPYGDTTAQNRPDFKQNNGKNP 300

605 TAAYESQYQEQYALWMGPYSVGKTVKASDRLMVDFSDITPYGDTTAQNRPDFKQNNGKNP 300

1053 TAAYESQYQEQYALWMGPYSVGKTVKASDRLMVDFSDITAYGDTTAQNRPDFKQNNGKNP 300

855 TAAYESQYQEQYALWMGPYSVGKTVKASDRLMVDFSDITPYGDTTAQNRPDFKQNNGKNP 300

1388 TAAYESQYQEQYALWMRPYSVGKTVKASDRLMVDFSDITPYGDTTAQNRPDFKQNNGKNP 300

362 TAAYESQYQEQYALWMGPYSVGKTVKASDRLMVDFSDITPYGDTTAQNRPDFKQNNGKNP 300

438 TAAYESQYQEQYALWMGPYSVGKTVKASDRLMVDFSDITPYGDTTAQNRPDFKQNNGKNP 300

1017 TAAYESQYQEQYALWMGPYSVGKTVKASDRLMVDFSDITPYGDTTAQNRPDFKQNNGKNP 300

436 TAAYESQYQEQYALWMGPYSVGKTVKASDRLMVDFSDITPYGDTTAQNRPDFKQNNGKNP 300

1070 TAAYESQYQEQYALWMGPYSVGKTVKASDRLTVDFSDITPYGDTTAQNRPDFKQNNGKNP 300

92 TAAYESQYQEQYALWMGPYSVGKTVKASDRLMVDFSDITPYGDTTAQNRPDFKQNNGKNP 300

449 TAAYESQYQEQYALWMGPYSVGKTVKVSDRLMVDFSDITPYGDTTAQNRPDFKQNNGKNP 300

247 TAAYESQYQEQYALWMGPYSVGKTVKASDRLMVDFSDITAYGDTTAQNRPDFKQNNGKNP 300

88-FA1090 TAAYESQYQEQYALWMGPYSVGKTVKASDRLMVDFSDITPYGDTTAQNRPDFKQNNGKNP 300

90-AR205 TAAYESQYQEQYALWMGPYSVGKTVKASDRLMVDFSDITPYGDTTAQNRPDFKQNNGKNP 300

460 TAAYESQYQEQYALWMGPYSVGKTVKASDRLMVDFSDITPYGDTTAQNRPDFKQNNGKNP 300

2385 TAAYESQYQEQYALWMGPYSVGKTVKASDRLMVDFSDITPYGDTTAQNRPDFKQNNGKNP 299

193-P9-17 TAAYESQYQEQYALWMGPYSVGKTVKASDRLMVDFSDITPYGDTTAQNRPDFKQNNGKNP 299

458 TAAYESQYQEQYALWMGPYSVGKTVKASDRLMVDFSDITPYGDTTAQNRPDFKQNNGKNP 299

65 TAAYESQYQEQYALWTGPYKVSKTVKASDRLMVDFSDITPYGDTTAQNRPDFKQNNGKKP 300

9 TAAYESQYQEQYALWMGPYSVGKTVKASDRLMVDFSDITPYGDTTAQNRPDFKQNNGKNP 300

2102 TAAYESQYQEQYALWMGPYSVGKTVKASDRLMVDFSDITPYGDTTAQNRPDFKQNNGKNP 300

*.:****** ***** **.*.****. * * ******* ******************:

1467 DVGNEVIRRRKGG 313

251 DVGNEVIRRRKGG 313

475 DVGNEVIRRRKGG 313

196 DVGNEVIRRRKGG 313

1471 DVGNEVIRRRKGG 313

464 DVGNEVIRRRKGG 313

439 DVGNEVIRRRKGG 313

446 DVGNEVIRRRKGG 313

2501 DVGNEVIRRRKGG 312

822 DVGNEVIRRRKGG 312

472 DVGNEVIRRRKGG 312

2657 DVGNEVIRRRKGG 313

2369 DVGNEVIRRRKGG 313

2143 DVGNEVIRRRKGG 313

2665 DVGNEVIRRRKGG 313

1048 DVGNEVIRRRKGG 313

2477 DVGNEVIRRRKGG 313

1047 DVGNEVIRRRKGG 313

1238 DVGNEVIRRRKGG 313

452 DVGNEVIRRRKGG 313

456 DVGNEVIRRRKGG 313

136 DVGNEVIRRRKGG 313

2077 DVGNEVIRRRKGG 313

1201 DVGNEVIRRRKGG 313

2176 DVGNEVIRRRKGG 313

2175 DVGNEVIRRRKGG 313

2702 DVGNEVIRRRKGG 313

2653 DVGNEVIRRRKGG 313

2604 DVGNEVIRRRKGG 313

2603 DVGNEVIRRRKEG 313

2599 DVGNEVIRRRKGG 313

2496 DVGNEVIRRRKGG 313

2375 DVGNEVIRRRKGG 313

2347 DVGNEVIRRRKGG 313

2170 DVGNEVIRRRKGG 313

2152 DVGNEVIRRRKGG 313

2083 DVGNEVIRRRKGG 313

1498 DVGNEVIRRLKGG 313

1481 DVGNEVIRRRKGG 313

1468 DVGNEVIRRRKGG 313

1459 DVGNEVIRRRKGG 313

1412 DVGNEVIHRRKGG 313

1296 DVGNEVIRRRKGG 313

1295 DVGNEVIRRRKGG 313

1202 DVGNEVIRRRKGG 313

1125 DVGNEVIRRRKGG 313

1057 DVGNEVIRRRKGG 313

678 DVGNEVIRRRKGG 313

582 DVGNEVIRRRKGG 313

469 DVGNEVIRRRKGG 313

466 DVGNEVIRRRKGG 313

462 DVGNEVIRRRKGG 313

459 DVGNEVIRRRKGG 313

437 DVGNEVIRRRKGR 313

431 DVGNEVIRRRKGG 313

2367 DVGNEVIRRRKGG 313

2326 DVGNEVIRRRKGG 313

2283 DVGNEVIRRRKGG 313

2282 DVGNEVIRRRKGG 313

1456 DVGNEVIRRRKGG 313

1242 DVGNEVIRRRKGG 313

1055 DVGNEVIRRRKGG 313

1054 DVGNEVIRRRKGG 313

789 DVGNEVIRRRKGG 313

600 DVGNEVIRRRKGG 313

455 DVGNEVIRRRKGG 313

451 DVGNEVIRRRKGG 313

2337 DVGNEVIRRRKGG 313

2089 DVGNEVIRRRKGG 313

468 DVGNEVIRRRKGG 313

442 DVGNEVIRRRKGG 313

2040 DVGNEVIRRRKGG 313

1075 DVGNEVIRRRKGG 313

521 DVGNEVIRRRKGG 313

2305 DVGNEVIRRRKGG 313

1473 DVGNEVIRRRKGG 313

1284 DVGNEVIRRRKGG 313

679 DVGNEVIRRRKGG 313

2146 DVGNEVIRRRKGG 313

1046 DVGNEVIRRRKGG 313

605 DVGNEVIRRHKGG 313

1053 DVGNEVIRRRKGG 313

855 DVGNEVIRRRKGG 313

1388 DVGNEVIRRRKGG 313

362 DVGNEVIRRRKGG 313

438 DVGNEVIRRRKGG 313

1017 DVGNEVIRRRKGG 313

436 DVGNEVIRRRKGG 313

1070 DVGNEVIRRRKGG 313

92 DVGNEVIRRRKGG 313

449 DVGNEVIRRRKGG 313

247 DVGNEVIRRRKGG 313

88-FA1090 DVGNEVIRRRKGG 313

90-AR205 DVGNEVIRRRKGG 313

460 DVGNEVIRRRKGG 313

2385 DVGNEVIRRKGG- 311

193-P9-17 DVGNEVIRRRKGG 312

458 DVGNEVIRRRKGG 312

65 DVGNEVIRRRKGG 313

9 DVGNEVIRRRKGG 313

2102 DVGNEVIRRRKGG 313

*******:*

Individual amino acid changes from the consensus at each position are highlighted in green
